# Anti‐CTLA‐4 m2a Antibody Exacerbates Cardiac Injury in Experimental Autoimmune Myocarditis Mice By Promoting Ccl5‐Neutrophil Infiltration

**DOI:** 10.1002/advs.202400486

**Published:** 2024-07-08

**Authors:** Ming‐Ming Wu, Yan‐Chao Yang, Yong‐Xu Cai, Shuai Jiang, Han Xiao, Chang Miao, Xi‐Yun Jin, Yu Sun, Xin Bi, Zi Hong, Di Zhu, Miao Yu, Jian‐Jun Mao, Chang‐Jiang Yu, Chen Liang, Liang‐Liang Tang, Qiu‐Shi Wang, Qun Shao, Qing‐Hua Jiang, Zhen‐Wei Pan, Zhi‐Ren Zhang

**Affiliations:** ^1^ Departments of Cardiology and Critical Care Medicine The First Affiliated Hospital of Harbin Medical University (HMU) NHC Key Laboratory of Cell Transplantation Key Laboratories of Education Ministry for Myocardial Ischemia Mechanism and Treatment Harbin 150001 China; ^2^ Departments of Cardiology and Pharmacy HMU Cancer Hospital Insitute of Metabolic Disease Heilongjiang Academy of Medical Science Heilongjiang key laboratory for Metabolic disorder and cancer related cardiovascular diseases Harbin 150081 China; ^3^ State Key Laboratory of Frigid Zone Cardiovascular Diseases (SKLFZCD) HMU Harbin 150081 China; ^4^ School of Interdisciplinary Medicine and Engineering HMU Harbin 150081 China; ^5^ Department of Pharmacology (State‐Province Key Laboratories of Biomedicine‐Pharmaceutics of China) Key Laboratory of Cardiovascular Medicine Research Ministry of Education) HMU Harbin 150081 China

**Keywords:** CTLA‐4, Cxcl1, myocarditis, neutrophil, spatial transcriptomics

## Abstract

The risk for suffering immune checkpoint inhibitors (ICIs)‐associated myocarditis increases in patients with pre‐existing conditions and the mechanisms remain to be clarified. Spatial transcriptomics, single‐cell RNA sequencing, and flow cytometry are used to decipher how anti‐cytotoxic T lymphocyte antigen‐4 m2a antibody (anti‐CTLA‐4 m2a antibody) aggravated cardiac injury in experimental autoimmune myocarditis (EAM) mice. It is found that anti‐CTLA‐4 m2a antibody increases cardiac fibroblast‐derived C‐X‐C motif chemokine ligand 1 (Cxcl1), which promots neutrophil infiltration to the myocarditic zones (MZs) of EAM mice via enhanced Cxcl1‐Cxcr2 chemotaxis. It is identified that the C–C motif chemokine ligand 5 (Ccl5)‐neutrophil subpopulation is responsible for high activity of cytokine production, adaptive immune response, NF‐κB signaling, and cellular response to interferon‐gamma and that the Ccl5‐neutrophil subpopulation and its‐associated proinflammatory cytokines/chemokines promoted macrophage (Mφ) polarization to M1 Mφ. These altered infiltrating landscape and phenotypic switch of immune cells, and proinflammatory factors synergistically aggravated anti‐CTLA‐4 m2a antibody‐induced cardiac injury in EAM mice. Neutralizing neutrophils, Cxcl1, and applying Cxcr2 antagonist dramatically alleviates anti‐CTLA‐4 m2a antibody‐induced leukocyte infiltration, cardiac fibrosis, and dysfunction. It is suggested that Ccl5‐neutrophil subpopulation plays a critical role in aggravating anti‐CTLA‐4 m2a antibody‐induced cardiac injury in EAM mice. This data may provide a strategic rational for preventing/curing ICIs‐associated myocarditis.

## Graphical abstract

This schematic presentation depicts anti‐CTLA‐4 m2a antibody increases the number of myocarditic zones (MZs) and worsens inflammatory responses and macrophage polarization, cardiac injury, and fibrosis in MZs of EAM, via the Cxcl1 (cardiac fibroblasts)/Cxcr2 (neutrophils) axis‐mediated increase in infiltration of Ccl5‐neutrophil subpopulation.

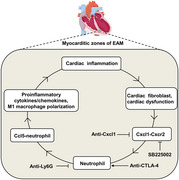

## 1 Introduction

Immune checkpoint inhibitors (ICIs) are monoclonal antibodies that target checkpoint proteins expressing in either immune cells or tumor cells. ICIs have achieved substantial clinical success and shown a significant survival benefit for several advanced malignancies. However, ICIs cause myocarditis with an incidence of 0.5–3.3% and a mortality rate of up to ≈50%.^[^ ^1^ ^]^ In addition, the risk of ICIs‐associated myocarditis, in patients older than 75 years and/or those with preexisting coronary artery disease, was significantly increased.^[^ ^2^ ^]^ More importantly, a population‐based observational study revealed that ICIs led to an almost two‐fold higher risk for developing cardiovascular events in patients with pr‐existing autoimmune disease (AD), in which myocarditis was the major event.^[^ ^3^ ^]^

Myocarditis is an inflammatory disease of the myocardium with infiltration of immune cells. The predominant infiltrated T cells and macrophages in the cardiac tissue have been characterized as one of the pathological signatures of ICIs‐associated myocarditis.^[^ ^1^, ^4^ ^]^ Previous studies show that T cells‐specific cytotoxic *T* lymphocyte antigen 4 (CTLA‐4) conditional knockout (cKO) mice exhibited severe systemic inflammatory phenotypes including myocarditis at four weeks after birth; interestingly, inducible CTLA‐4 KO in adult mice also caused systemic inflammatory phenotypes without signs of myocarditis.^[^ ^5^ ^]^ These results suggest that deletion of the CTLA‐4 gene in different developmental period of the mice causes separate features of pathological processes and the discrepancy in the organs. A recent study showed that the application of high doses programmed cell death protein 1 (PD‐1) inhibitor and CTLA‐4 inhibitor caused significant systemic inflammation and multiorgan immune‐related adverse events including myocarditis in cynomolgus monkeys.^[^ ^6^ ^]^ Furthermore, PD‐1 deficiency resulted in the development of fatal myocarditis in MRL/MpJ‐Faslpr mice, which was associated with considerable infiltration of T cells, myeloid cells, and autoantibodies against cardiac α‐myosin.^[^ ^7^ ^]^

Cardiac α‐myosin is a potent heart‐specific autoantigen and can be recognized by autoantibodies in patients with myocarditis and dilated cardiomyopathy.^[^ ^8^ ^]^ Recent study showed that peripheral blood *T*‐cells were expanded by α‐myosin peptides and these α‐myosin‐expanded *T*‐cells shared TCR clonotypes with inflamed cardiac and skeletal muscle cells in patients with ICI‐associated myocarditis.^[^ ^9^ ^]^ Immunization of BALB/c mice with cardiac a‐myosin peptide caused experimental autoimmune myocarditis (EAM), having a significantly increased level of circulating heart myosin autoantibody.^[^ ^10^ ^]^ Indeed, infiltration of neutrophils in the heart was strongly associated with the severity of acute viral myocarditis^[^ ^11^ ^]^ and depletion of neutrophils in the early acute phase improved viral myocarditis‐induced cardiac necrosis by reducing cardiac monocyte recruitment and proinflammatory macrophage differentiation.^[^ ^12^ ^]^ However, at the present time, there is no evidence on whether neutrophils play a role in ICIs‐induced myocarditis. Therefore, we used the EAM mouse model to decipher whether and how anti‐CTLA‐4 m2a antibody exacerbates the EAM‐induced cardiac injury.

We found that injection of anti‐CTLA‐4 m2a antibody alone did not cause any feasible alteration in the hearts of wild‐type mice (control mice). We identified, for the first time, that Ccl5‐neutrophil subpopulation‐mediated increase in the production of cytokines, chemokines, and macrophage M1 polarization play the dominant role in anti‐CTLA‐4 m2a antibody‐induced cardiac injury in EAM mice. We further revealed that the Cxcl1‐Cxcr2 axis was responsible for recruiting neutrophils into the MZ of EAM mice and that anti‐CTLA‐4 m2a antibody exacerbated Cxcl1‐Cxcr2 axis‐mediated infiltration of Ccl5‐neutrophil subpopulation. Finally, depletion of neutrophils attenuated cardiac inflammation and dysfunction in both anti‐IgG antibody‐ and anti‐CTLA‐4 m2a antibody‐treated EAM mice. The data suggest that the Ccl5‐neutrophil subpopulation may be a potential interventional target for preventing ICIs‐associated myocarditis.

## 2 Results

### 2.1 Anti‐CTLA‐4 m2a Antibody Aggravates Inflammation‐Mediated Cardiac Dysfunction in EAM Mice

**Figure 1:**
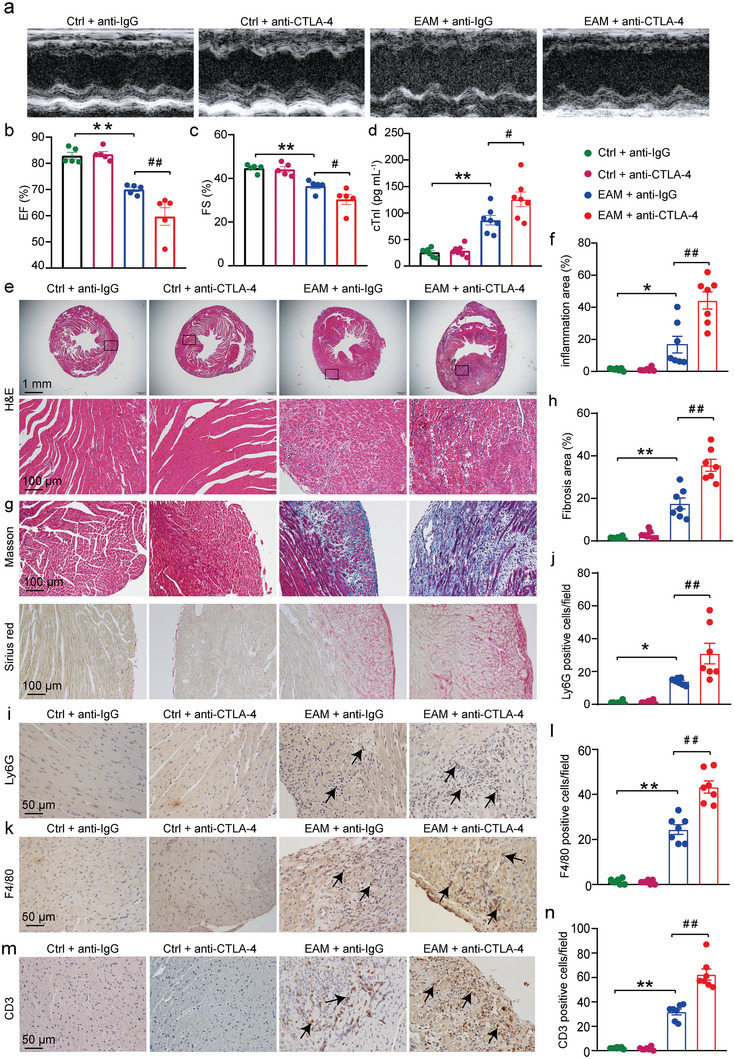
Anti‐CTLA‐4 m2a antibody aggravates cardiac inflammation and dysfunction in EAM mice. a–c) Representative M‐mode view of echocardiography obtained from each indicated experimental group a). Summarized mean data of ejection fraction (EF) b) and fractional shortening (FS) c) from each experimental group (*n* = 5 for each group). d) The data examined by an ELISA kit demonstrating the cTnI serum levels obtained from each indicated experimental group (*n* = 7 for each group). e,f) Representative H&E staining of the cardiac sections (the top panel; Scale bar: 1 mm) and (the bottom panel; Scale bar: 100 µm) from each indicated group e) and the quantified data f) demonstrating the inflammation areas (%, H&E staining images analyzed by ImageJ Software) under the indicated conditions (*n* = 7 for each group) g,h)The representative images of Masson (upper panel) and Sirius Red (lower panel) staining of the heart sections from each indicated group g); the quantified data presenting cardiac fibrosis areas (%, Masson staining analyzed by ImageJ Software) under the different experimental conditions h) (*n* = 7 for each group). i–n) Representative immunostaining demonstrating infiltration of neutrophils (Ly6G positive) i), macrophages (F4/80 positive) k), and *T*‐cells (CD3 positive) m) in cardiac sections generated from each experimental group (scale bars equal 50 µm). Quantification data showing the absolute number of neutrophils j), macrophages l), and *T*‐cells n) per field (*n* = 7 for each group; determined by ImageJ software). Data are presented as the mean ± SEM. Statistics: One‐way ANOVA followed by Tukey's post hoc multiple comparisons test was used for analyzing **b**, **c**, **d**, **f**, **h**, **j**, **l**, and **n**. The statistics were performed between indicated groups; ^*^ and ^#^ indicate *p <* 0.05; ^**^ and ^##^ represent *p <* 0.01. Hereafter, Ctrl + anti‐IgG antibody, Ctrl + anti‐CTLA‐4 m2a antibody, EAM + anti‐IgG antibody, and EAM + anti‐CTLA‐4 m2a antibody respectively represent anti‐IgG antibody‐treated control mice group, anti‐CTLA‐4 m2a antibody‐treated control mice group, anti‐IgG antibody‐treated EAM mice group, anti‐CTLA‐4 m2a antibody‐treated EAM mice group.

Previous study showed that inflammation peaks on days 14–21 after establishing the EAM model;^[^ ^13^ ^]^ therefore, EAM mice were intraperitoneally injected with either 10 mg kg^1^ anti‐mouse CTLA‐4 m2a antibody or 10 mg kg^1^ anti‐rat IgG antibody (served as control) on day 14, 16, 18, 20 and the animals were sacrificed on day 21 after initial immunization (Figure S1a, Supporting Information). The data showed that neither anti‐IgG antibody nor anti‐CTLA‐4 m2a antibody caused cardiac dysfunction and other pathological alterations in the hearts (**Figure** **1**a–g). Injection of α‐myosin led to significant cardiac dysfunction (Figure 1a–c), concomitant with the increased serum cardiac troponin I (cTnI) levels (Figure 1d) and infiltration of immune cells as reflected by the inflammation area in the cardiac tissue (Figure 1e,f) and cardiac fibrosis (Figure 1g,h). Importantly, these alterations in EAM mice were dramatically aggravated by the application of anti‐CTLA‐4 m2a antibody (Figure 1a–h). Masson and Sirius red staining showed that anti‐CTLA‐4 m2a antibody further exacerbated myocardial fibrosis and collagen deposition in the myocardium of EAM mice compared with anti‐IgG antibody‐treated EAM mice (Figure 1g,h). In addition, the data demonstrated that the application of anti‐CTLA‐4 m2a antibody further increased the infiltration of neutrophils (Ly6G^+^), macrophages (F4/80^+^), and *T*‐cells (CD3^+^) in the cardiac tissue of EAM mice (Figure 1i–n). These data suggest that the anti‐CTLA‐4 m2a antibody promotes immune cell infiltration and cardiac dysfunction in EAM mice.

### 2.2 Anti‐CTLA‐4 m2a Antibody Leads to the Heterogeneity of Infiltrated Neutrophils in the Cardiac Tissue of EAM Mice

**Figure 2:**
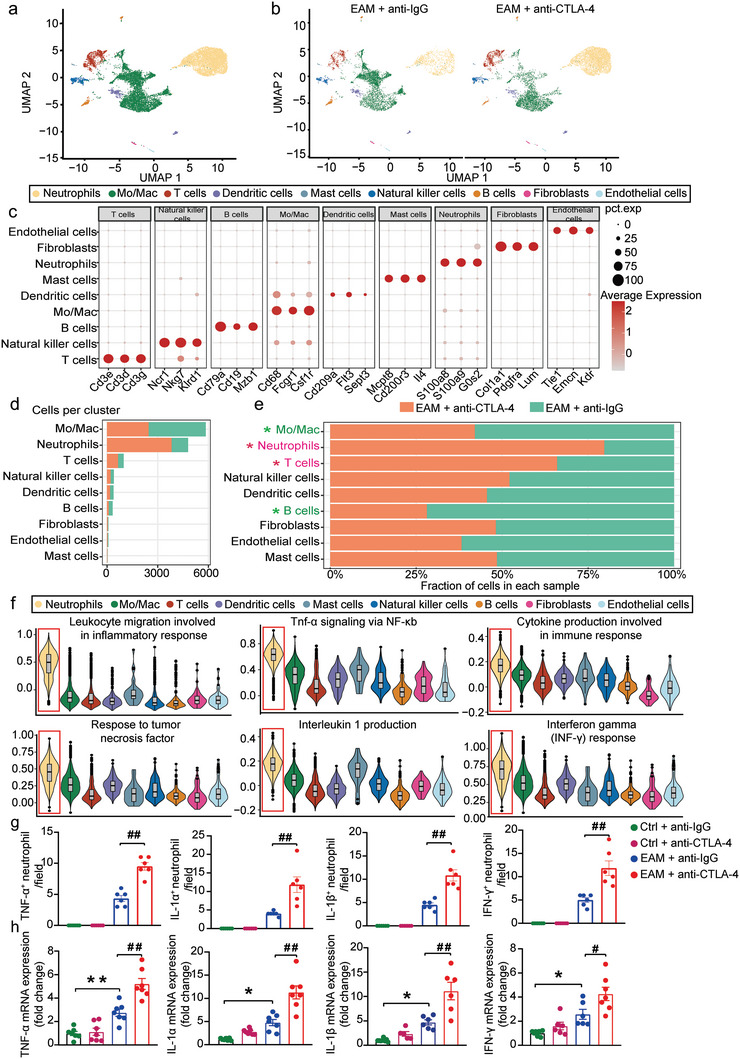
Identification of the cell types in mouse hearts using scRNA‐seq. a) UMAP plot showing that there were nine cell types associated clusters from both the anti‐IgG antibody‐ and anti‐CTLA4 m2a antibody‐treated EAM mouse hearts and each point presenting a single cell marked with different colors according to cluster designation. b) UMAP plots demonstrating the discrepancy of cardiac cell clusters between anti‐IgG antibody‐ and anti‐CTLA4 m2a antibody‐treated EAM mice. c) Dot plots representing the expression profiles of representative marker genes of each identified cell type. Circle size indicates the percentage of cells expressing marker genes, and dot color indicates the average expression levels of the marker genes in the designated cell type. d,e) The absolute numbers of each cell type from the sorted CD45^+^ leukocytes of the hearts d) and the relative proportion of each cell type scaled by the total number of cells per condition e); the colored bars in green and in orange respectively demonstrating the alterations in each indicated cell type between anti‐IgG antibody‐ and anti‐CTLA‐4 m2a antibody‐treated EAM mice. *p*‐value was calculated by chi‐square test (the red asterisks indicate a significant increase; the green asterisk represents a significant decrease). f) Violin plots statistically compare enrichment scores of genes annotated for relative pathways in each cardiac cell type. g) Quantitative analyses demonstrating the number of Tnf‐α^+^ neutrophils, Il‐1α^+^ neutrophils, Il‐1β^+^ neutrophils and INF‐γ^+^ neutrophils in the hearts from each indicated experimental group. (*n* = 6 for each group). h) The relative gene expression of TNF‐α, Il‐1α, Il‐1β, and INF‐γ analyzed by qPCR in mouse hearts from each experimental group (*n* = 5–8 for each group). Data are presented as the mean ± SEM. Statistics: unpaired two‐tailed Student's *t*‐tests and one‐way ANOVA, followed by Tukey's post hoc multiple comparisons test were respectively used for analyzing g and h. The statistics were performed between indicated groups; ^*^ and ^#^ indicate *p <* 0.05; ^**^ and ^##^ represent *p <* 0.01.

To delineate the immune repertoire of anti‐CTLA‐4 m2a antibody‐associated myocarditis in the peak inflammatory phases, we performed flow cytometry experiments to collect live cardiac CD45^+^ leukocytes on day 21 post‐initial immunization and subjected the cells to single‐cell RNA sequencing (scRNA‐seq) (Figure S1d,e, Supporting Information). After quality control, 6427 and 8065 cells were obtained from anti‐IgG‐treated group and anti‐CTLA‐4 m2a antibody‐treated group, respectively (Figure S1f, Supporting Information). Unsupervised clustering identified 9 major clusters, and cell identities were validated by the expression of cell‐specific marker genes (**Figure** **2**a–c; Figure S2, Supporting Information). Among which, the most abundant cell types were monocytes/macrophages and neutrophils (Figure 2d). To analyze whether anti‐CTLA‐4 m2a antibody may alter the landscape of these cell populations in the cardiac tissue of EAM mice, the relative proportions of each cell type in both groups were analyzed as previously described.^[^ ^14^ ^]^ The data showed that the proportions of neutrophils and *T*‐cells were significantly increased (the pink font with asterisk), whereas the proportions of monocytes/macrophages and B cells (the green font with asterisk) were significantly decreased by anti‐CTLA‐4 m2a antibody in EAM mice (Figure 2e).

*AddModuleScore* function of the Seurat package was used to further investigate the main sources of cytokine/chemokine production and inflammatory; cytokine scores in each identified cell type were calculated. Our data showed that among the identified cell types within the cardiac tissue, the neutrophil had the highest inflammatory cytokine scores (Figure 2f; indicated by the red squares respectively). Furthermore, co‐immunofluorescent staining revealed that the number of Tnf‐α^+^‐, Il‐1α^+^‐, Il‐1β^+^‐ and IFN‐γ^+^‐neutrophils was significantly increased by anti‐CTLA‐4 m2a antibody in the cardiac tissues of EAM mice (Figure 2g;Figure S3a–d, Supporting Information). Consistently, our qRCR results showed that the mRNA levels of Tnf‐α, Il‐1α, Il‐1β, and IFN‐γ were also significantly increased by anti‐CTLA‐4 m2a antibody in the cardiac tissues of EAM mice (Figure 2h). In addition, among the four identified *T*‐cell subpopulations, the proportion of CD4 Treg cells was significantly increased, and the weight of Th17 like cells was significantly decreased by anti‐CTLA‐4 m2a‐antibody in EAM mouse hearts. The number of infiltrated CD8^+^ effector *T*‐cells was not significantly altered by anti‐CTLA‐4 m2a‐antibody in EAM mouse hearts (Figure S4a–d, Supporting Information). Moreover, lymphocyte proliferation and regulation of *T*‐cell activation signaling pathways in the CD8^+^ effector *T*‐cells were inhibited by anti‐CTLA‐4 m2a‐antibody in EAM mice, as revealed by DEGs and performed GO enrichment analyses (Figure S4 e,f, Supporting Information). These data suggest that the increased infiltration of neutrophils and neutrophil‐mediated enhancement of cytokine/chemokine production, but not *T*‐cells, were responsible for worsening CTLA‐4 m2a antibody‐associated cardiac inflammation and dysfunction in EAM mice.

### 2.3 Anti‐CTLA‐4 m2a Antibody‐Induced Infiltration of Ccl5‐Neutrophil Subpopulation Exacerbates Cardiac Inflammation in EAM Mice

**Figure 3:**
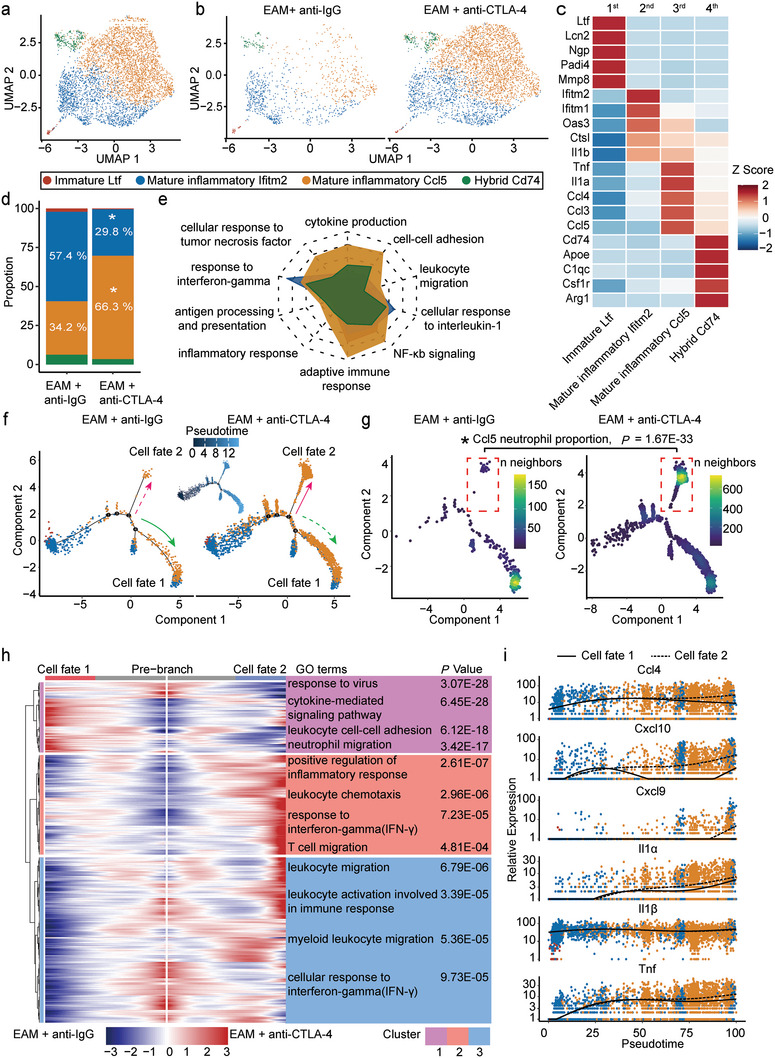
Anti‐CTLA‐4 m2a antibody‐mediated increase in the infiltration of Ccl5‐neutrophil subpopulation in the hearts of EAM mice. a) UMAP plot showing four neutrophil subpopulations (immature Ltf‐neutrophil, mature inflammatory Ifitm2‐neutrophil, mature inflammatory Ccl5‐neutrophil, and Hybrid Cd74‐neutrophil) colored by manually annotated clusters. b) UMAP plots demonstrating the differences of infiltrated neutrophil subpopulations in the hearts between anti‐IgG antibody‐ and anti‐CTLA‐4 m2a antibody‐treated EAM mice. c) Heatmap showing the relative expression (as reflected by z score) levels of marker genes in each subpopulation of neutrophils. d) Bar graph, colored according to cluster designation, showing the proportions of each neutrophil subpopulation relative to all cardiac neutrophils in anti‐IgG antibody‐ and anti‐CTLA‐4 m2a antibody‐treated EAM mice. *p* value was calculated by chi‐square test and the white asterisks indicated the significant differences. e) Radar plot showing GO enrichment of related proinflammatory and cytokine signaling in four neutrophil subpopulations. f) Pseudotime analyses demonstrating cell trajectories differed between anti‐IgG antibody‐ and anti‐CTLA‐4 m2a antibody‐treated EAM mice and differentiated toward to cell fate 1 and cell fate 2. g) Pseudotime analyses respectively demonstrating the relative neighborhood density of the Ccl5‐neutrophil subpopulation, as labeled by the red dashed rectangular square. *p*‐value was calculated by chi‐square test; ^*^ indicates *p* = 1.67E‐33. h) BEAM heatmap representing the expression profiles of branch‐dependent genes over pseudotime. The branch point (in the middle of the heatmap) is the beginning of pseudotime. Both sides of the heatmap are the ends of pseudotime. Ifitm2‐and Ccl5‐neutrophil subpopulation‐related marker genes are enriched in cells on cell fate 1 trajectory (anti‐IgG antibody), and the Ccl5‐neutrophil subpopulation related marker genes are mainly enriched in cells on cell fate 2 trajectory (anti‐CTLA‐4 m2a antibody). Accordingly, genes are clustered into three modules based on expression patterns across development, in which the important GO terms are related to biological processes. i) Pseudotime analyses, from the root of the trajectory, demonstrating the dynamic alterations of genes (Ccl4, Cxcl10, Cxcl9, Il‐1α, Il‐1β, and Tnf) during differentiating to cell fate 1 (the solid line) or cell fate 2 (dashed line); the different colored dots indicating the identities of neutrophil subpopulations and each dot representing a single cell.

Utilizing the classical markers, we identified four distinct neutrophil subpopulations in both anti‐IgG antibody‐ and anti‐CTLA‐4 m2a antibody‐treated EAM mice (**Figure** **3**a–c). The first cluster was consisted of a few “immature” neutrophils expressing Ltf, Lcn2, Ngp, Padi4, and MMP8 (the first panel of Figure 3c); the second cluster was consisted of “inflammatory mature” neutrophils expressing IFN‐induced genes (ifitm1 and ifitm2) and Oas3, which can modulate inflammation (the second panel of Figure 3c); the third population was inflammatory mature neutrophils expressing high levels of cytokines (Tnf and Il‐1α) and leukocyte chemotaxis (Ccl3, Ccl4, and Ccl5) (the third panel of Figure 3c); the final subset was characterized as “hybrid” neutrophils due to their macrophage‐like characteristics, i.e., complement activation genes (C1qc and Cd74), Apoe and Arg1 (the fourth panel of Figure 3c). Interestingly, the proportion of inflammatory mature Ccl5‐neutrophil subpopulation was significantly increased (34.2% vs 66.3%), and the ratio of Ifitm2‐neutrophil subpopulation was significantly decreased (54.7% vs 29.8%) by application of anti‐CTLA‐4 m2a‐antibody in EAM mice (Figure 3d). By comparing the overall activity of the four neutrophil subpopulations and calculating the significant cytokine signaling pathway‐associated gene set enrichment scores, we found that Ccl5‐neutrophil subpopulation exhibited the highest immune and inflammatory activities including cytokine production, cellular response to Tnf, adaptive immune response and NF‐κb signaling, *etc*. (Figure 3e; indicated by the orange area), and that Ifitm2‐neutrophils scored slightly higher in response to interferon gamma and cellular response to interleukin 1 (Figure 3e; indicated by the blue area).

We next analyzed the expression profile of the three different neutrophil subpopulations (the hybrid Cd74‐neutrophil was discarded as a doublet), using pseudotime trajectory analysis with Monocle. The data showed that there were three major different cell fates of neutrophils (Figure 3f). The density curves of cell subpopulation expression generated from the trajectory showed that the Ltf‐neutrophil subpopulation was mainly localized at the initiating branch of the trajectory and developed into two opposite differentiation directions (two distinct cell fates) and that the two termini of the trajectory were designated as fate 1 (Ifitm2‐neutrophil and Ccl5‐neutrophil subpopulations) and fate 2 (Ccl5‐neutrophil subpopulation) (Figure S5, Supporting Information). Furthermore, the majority of Ltf‐neutrophil subpopulations were differentiated toward cell fate 1 at the lower trajectory path in anti‐IgG antibody treated EAM mice (Figure 3f; the solid green arrowhead in the left panel). In contrast, anti‐CTLA‐4 m2a antibody promoted differentiation of Ltf‐neutrophil subpopulations toward both the cell fate 2 (the upper trajectory path marked by the solid pink arrowhead in the right panel) and fate 1 at the lower trajectory path (Figure 3f; the dashed green arrowhead in the right panel). More importantly, the application of anti‐CTLA‐4 m2a antibody in EAM mice skewed the differentiation of neutrophils toward Ccl5‐neutrophil subpopulation of the fate 2 branch compared to that of the anti‐IgG antibody‐treated EAM mice (the upper trajectory paths highlighted by the dashed red squares) (Figure 3g). The branch expression analysis modeling (BEAM) and pseudotime heatmaps revealed that the expression of genes is dynamic along directions and pseudotime, respectively. Based on the transcriptional changes associated with transitional states, three different gene expression modules with different immune/inflammatory pathways were identified (modules 1–3) (Figure 3h). In addition, pseudotime kinetic analyses revealed that the chemokines (Ccl4, Cxcl10, and Cxcl9) and cytokines (Il‐1α, Il‐1β, and Tnf) gradually increased, during the differentiation from the immature Ltf‐neutrophil to Ccl5‐neutraphil (fate 2, marked with the orange dots, the dashed black lines) and Ifitm2‐neutrophil (fate 1, marked with the blue dots, the solid black lines) (Figure 3i). Moreover, the expression levels of Ccl4, Cxcl10, Cxcl9, Il‐1α, and Tnf in Ccl5‐neutrophil subpopulation were much higher than those in Ifitm2‐neutrophil subpopulation upon application of anti‐CTLA‐4 m2a antibody (Figure 3i). These results together suggest that the mature inflammatory Ccl5‐neutrophil subpopulation‐mediated increase in cytokine/chemokine production is responsible for exacerbating cardiac inflammation in anti‐CTLA‐4 m2a antibody‐treated EAM mice.

### 2.4 Anti‐CTLA‐4 m2a Antibody Promotes M1 Macrophage Polarization in the Cardiac Tissue of EAM Mice

**Figure 4:**
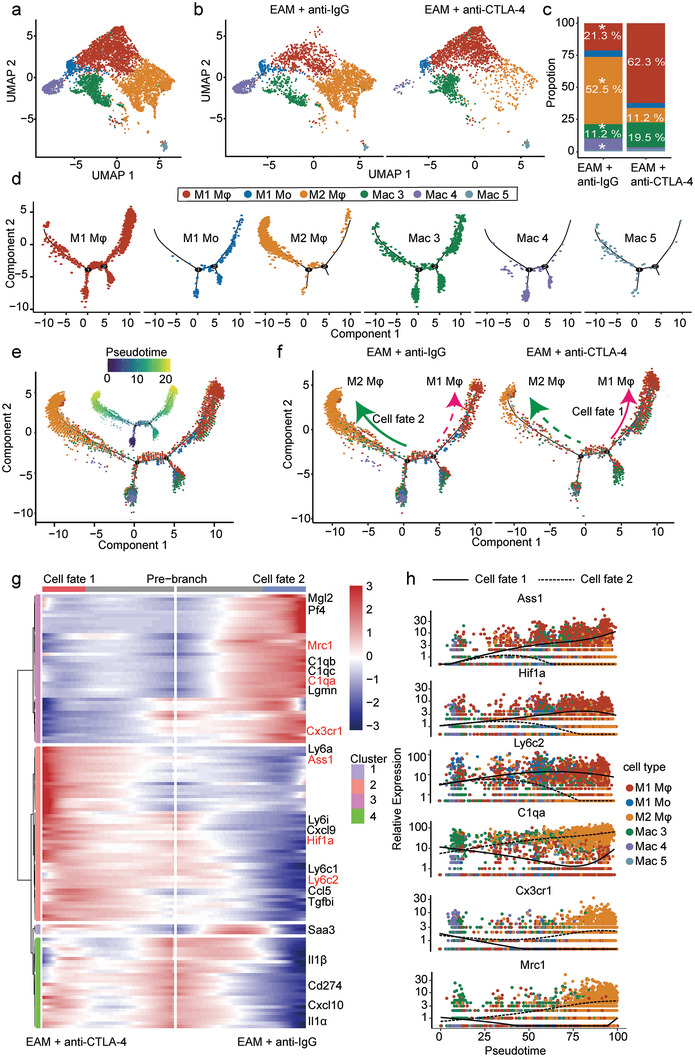
Anti‐CTLA‐4 m2a antibody promotes M1 macrophage polarization in EAM mice. a) UMAP plot showing six macrophage subpopulations (M1 Mφ, M1 Mo, M2 Mφ, MaC 3, MaC 4, and MaC 5) colored by manually annotated clusters. b) UMAP plots demonstrating the differences of infiltrated monocyte/macrophage subpopulations in the hearts between anti‐IgG antibody‐ and anti‐CTLA‐4 m2a antibody‐treated EAM mice. c) Bar graph showing the proportions of each monocyte/macrophage subpopulation relative to all cardiac monocytes/macrophages in the anti‐IgG antibody‐ and anti‐CTLA‐4 m2a antibody‐treated EAM mice, colored according to cluster identity. *p* value was calculated by chi‐square test and the white asterisks indicated the significant differences. d) Monocle pseudotime trajectories showing the differentiation of M1 Mφ, M1 Mo, M2 Mφ, MaC 3, MaC 4, and MaC 5 macrophage subpopulations from both anti‐IgG antibody‐ and anti‐CTLA‐4 m2a antibody‐treated EAM mice. e) Pseudotime analysis revealed the cell trajectories within six macrophage subpopulations. f) Pseudotime analyses demonstrating cell trajectories differed between anti‐IgG antibody‐ and anti‐CTLA‐4 m2a antibody‐treated EAM mice. g) BEAM heatmap representing the expression profiles of the branch‐dependent genes over pseudotime. Genes are clustered into four modules based on expression patterns across pseudotime. The branch point shown in the middle of the heatmap is the beginning of pseudotime. Both sides of the heatmap are the ends of pseudotime. The colored bar, coding from the blue to the red, indicates the relative gene expression level from low to high (the upper bar). M1 Mφ and Mac 3 macrophage subpopulation‐related marker genes are enriched in cells on cell fate 1 of the trajectory, and M2 Mφ macrophage subpopulation related marker genes are enriched in cells on cell fate 2 of the trajectory. Genes are clustered into four modules based on expression patterns across development (the lower bar). h) Pseudotime analyses, from the root of the trajectory, demonstrating the dynamic alterations of genes (Ass1, Hif1α, Ly6c2, C1qa, Cx3cr1, and Mrc1) during differentiating to cell fate 1 (the solid line) or cell fate 2 (dashed line). Each dot represents a single cell and the dot color indicates each indicated neutrophil subpopulation respectively.

We further investigated the dynamics and functional discrepancy of monocytes/macrophages in the cardiac tissues between the anti‐IgG antibody‐ and anti‐CTLA4 m2a antibody‐treated EAM mice, according to the macrophage distribution and the markers described previously.^[^ ^15^ ^]^ Six distinct infiltrated monocyte/macrophage subpopulations (a minor cluster was discarded as a doublet) were identified (**Figure** **4**a,b), each subpopulation having a unique transcriptional profile as follows: a minor classical blood‐derived M1 monocytes (M1 Mo); a major subpopulation of classical monocyte‐derived M1 macrophages (M1 Mφ); a subpopulation of nonclassical M2 macrophages (M2 Mφ); the other three subpopulations were respectively defined as macrophage (Mac) 3, Mac 4 and Mac 5 (Figure S6, Supporting Information). Importantly, the proportions of M1 Mφ and Mac 3 were respectively increased from 21.3% to 62.3% and 11.2% to 19.5%, and the proportions of M2 Mφ and Mac 4 were respectively decreased from 52.5% to 11.2% and 8.9% to 1.3% by anti‐CTLA‐4 m2a antibody in the cardiac tissue of EAM mice (Figure 4c).

We performed pseudotime trajectory analysis to investigate the temporal dynamics and transitions among the infiltrated monocyte/macrophage subpopulations. The data showed that the proinflammatory M1 Mφ and Mac 3, predominantly from the anti‐CTLA‐4 m2a antibody‐treated EAM mouse hearts, straddled among all branches across the pseudotime trajectory and mainly located at the right‐side trajectory path (Figure 4d). Meanwhile, M2 Mφ and Mac 5 subpopulations, enriched in the anti‐IgG antibody‐treated EAM mice, were dominantly distributed at the left‐side trajectory path. M1 Mo started at the root of the pseudotime trajectory and mainly located at the right‐side trajectory path. Mac 4 preferentially occupied specific early branches of the trajectory (Figure 4d). More importantly, while M1 Mo mainly differentiated toward to M2 Mφ in anti‐IgG antibody‐treated EAM mice (Figure 4e,f; the solid green arrowhead in the left panel, cell fate 2), anti‐CTLA‐4 m2a antibody promoted differentiation of M1 Mo mainly toward to M1 Mφ and Mac 3 (Figure 4e,f; the solid pink arrowhead in the right panel, cell fate 1).

To further characterize the gene signature of monocyte/macrophage subpopulations distributed alongside the pseudotime trajectory, differentially expressed genes (DEGs) that covary alongside pseudotime were visualized by Monocle. The expression levels of M1 Mφ‐related signature genes (Ass1, Hif1a, and Ly6c2), enriched in anti‐CTLA4 m2a antibody‐treated EAM mouse hearts, were significantly increased at the left‐side of the pseudotime trajectory (Figure 4g; the second panel of the left side). M2 Mφ‐associated genes, including C1qa Cx3cr1 and Mrc1 (CD206), enriched in anti‐IgG antibody‐treated EAM mouse hearts, were greatly upregulated at the right‐side of the cell trajectory (Figure 4g; the first panel of the right side). Further analyses demonstrated that anti‐CTLA4 m2a antibody led to a significant increase in the expression levels of M1 Mφ‐related gene signatures including Ass1, Hif1α, and Ly6c2 (Figure 4h; the solid black line) and also resulted in a significant decrease in the expression levels of M2 Mφ‐related genes including C1qa, Cx3cr1, and Mrc1 (CD206) in EAM mouse hearts (Figure 4h; the dashed black line). These results suggest that anti‐CTLA4 m2a antibody‐mediated M1 macrophage polarization plays a critical role in exacerbating cardiac proinflammatory response in the EAM mice.

### 2.5 The Cxcl1‐Cxcr2 Axis Drives Infiltration of Neutrophils

Cell‐cell communication analyses of receptor‐ligand pair interactions were performed to explore the potential mechanisms by which anti‐CTLA‐4 m2a antibody increased neutrophil infiltration in the EAM mouse hearts. Cell–cell communications in both quantity (467 vs 452) and strength (0.44 vs 0.659) were respectively found in anti‐IgG antibody‐ and anti‐CTLA‐4 m2a antibody‐treated EAM mouse hearts (Figure S7a, Supporting Information). Moreover, we found that monocytes/macrophages and neutrophils were the major signaling sources and target biological communications in the anti‐IgG antibody‐ and anti‐CTLA‐4 m2a antibody‐treated EAM mouse hearts, respectively (Figure S7b,c, Supporting Information).

**Figure 5:**
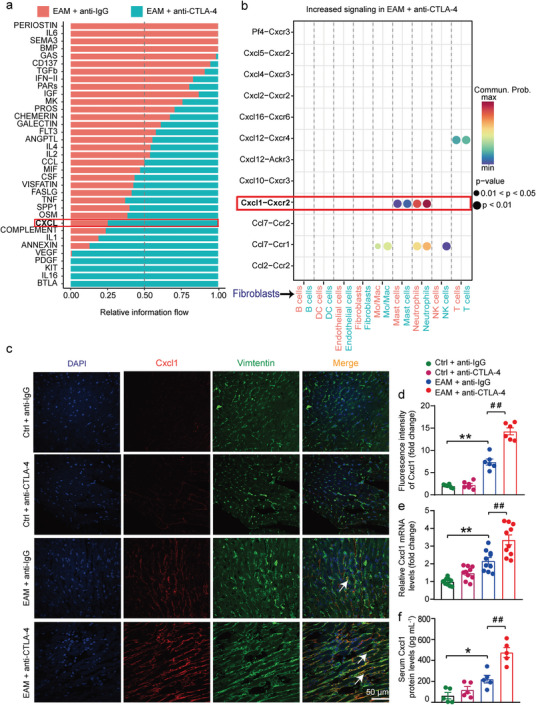
Cardiac fibroblast‐derived Cxcl1 and Cxcr2 of neutrophils promote neutrophil infiltration in the cardiac tissues of EAM mice. a) Bar graph showing the differences in the overall information flow of the significant signaling pathways between anti‐IgG antibody‐ and anti‐CTLA‐4 m2a antibody‐treated EAM mouse hearts. The top signaling pathways (the red) are more enriched in anti‐IgG antibody‐treated EAM mice, and bottom the pathways (the blue) are more enriched in anti‐CTLA‐4 m2a antibody‐treated EAM mice. CXCL signaling pathways were significantly increased by anti‐CTLA‐4 m2a antibody in EAM mice, as marked by the red solid rectangular square. b) Bubble plot showing the differentially expressed ligand‐receptor pairs between anti‐IgG antibody‐ and anti‐CTLA‐4 m2a antibody‐treated EAM mouse hearts and demonstrating the strength of increased signaling from cardiac fibroblasts to B cells, dendritic cells (DCs) endothelial cells (ECs), fibroblasts, monocytes/macrophages, master cells, neutrophils, nature killer cells (NK), and *T*‐cells. The highlighted Cxcl‐Cxcr2 signaling was significantly increased by anti‐CTLA‐4 m2a antibody in the mouse hearts compared with that in anti‐IgG antibody‐treated EAM mouse hearts, as labeled by the red solid rectangular square. The color (from the blue to red) and size of the dots represent the communication probability and *p* values (shown on the right), respectively. The empty spaces indicated that the communication probability was zero. *p* values are computed from a two‐sided permutation test. c) Representative immunofluorescence staining of Cxcl1 (red) and vimentin (green) expression in the MZs of mice from each indicated experimental group. The white arrows represent the colocalization of Cxcl1 with the cardiac fibroblast marker vimentin. Scale bars equal 50 µm. d,e) Quantitative analyses demonstrating the fluorescence intensity intensities of Cxcl1 (*n* =  6 for each group) d) and mRNA expression levels of Cxcl1 (*n* = 10 for each group) e) in the hearts from each indicated experimental group. f) ELISA experiments showing the serum Cxcl1 protein levels of each indicated experimental group (*n* = 5 for each group). Data are presented as the mean ± SEM. Statistics: one‐way ANOVA, followed by Tukey's post hoc multiple comparisons test was used for analyzing (d), (e), and (f). The statistics were performed between indicated groups; ^*^ indicates *p <* 0.05; ^**^ and ^##^ represent *p <* 0.01.

We examined the overall changes in each signaling pathway in the hearts between anti‐IgG antibody‐ and anti‐CTLA‐4 m2a antibody‐treated EAM mice. The CXCL signaling pathways in the heart were significantly increased by anti‐CTLA‐4 m2a antibody in EAM mice (**Figure** **5**a; highlighted by the red solid rectangular square). CellChat analyses revealed that among CXCL signaling pathways, the ligand‐receptor pair Cxcl1‐Cxcr2 was the most significant contributor to communication between cardiac fibroblasts and neutrophils (Figure 5b; highlighted by the red solid rectangular square). Immunofluorescent staining of cardiac sections demonstrated that the fluorescence intensity of Cxcl1 (the red staining) in cardiac fibroblasts (the green staining) was significantly increased by anti‐CTLA‐4 m2a antibody compared to that in anti‐IgG antibody‐treated EAM mice (Figure 5c,d). Moreover, the Cxcl1 protein levels in serum and mRNA levels in cardiac tissues were also significantly increased by anti‐CTLA‐4 m2a antibody compared to those in anti‐IgG antibody‐treated EAM mice (Figure 5e,f). These data suggest that the Cxcl1‐Cxcr2 axis, between cardiac fibroblasts (Cxcl1) and neutrophils (Cxcr2), drove neutrophil infiltration in the cardiac tissues of EAM mice and that the neutrophil recruitment in the cardiac tissues of EAM mice was further strengthened by anti‐CTLA‐4 m2a antibody.

**Figure 6:**
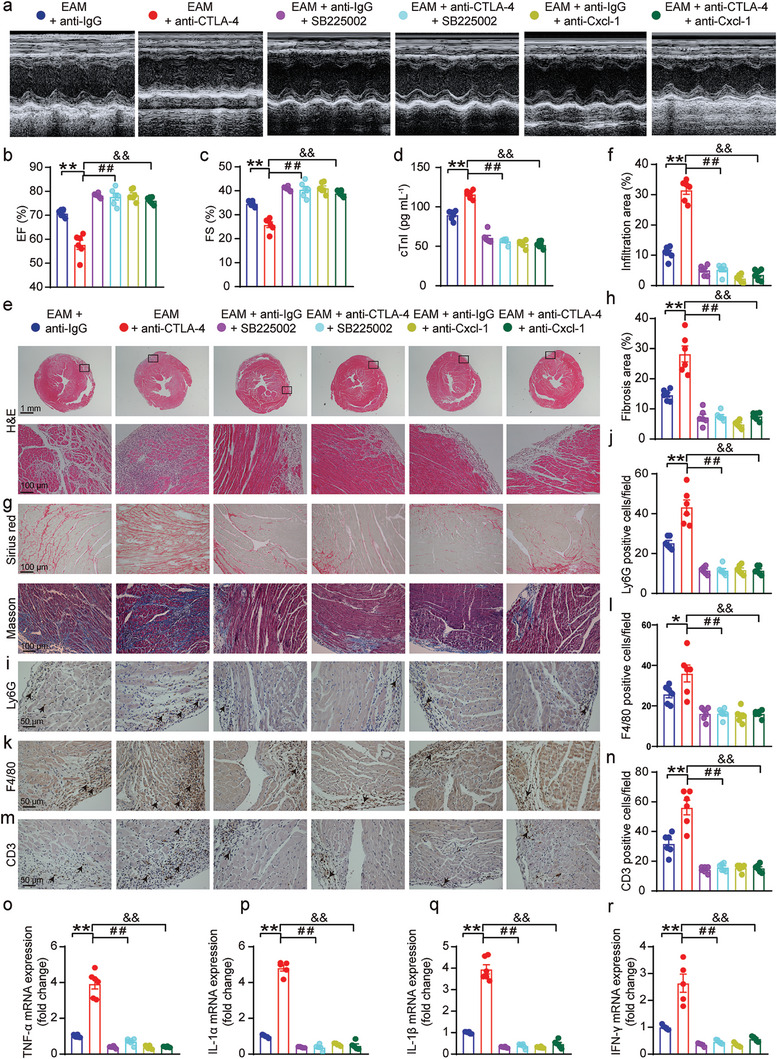
Selective Cxcr2 antagonist SB225002 or Cxcl1 neutralizing antibody ameliorated anti‐CTLA‐4 m2a antibody‐induced cardiac injury in EAM mice. a–c) Representative M‐mode view of echocardiography obtained from each indicated experimental group a); summarized mean data of ejection fraction (EF) b) and fractional shortening (FS) c) from each indicated group (*n* = 6 for each group). d) The data examined by an ELISA kit demonstrating the CTnI serum levels obtained from each indicated experimental group (*n* = 6 for each group). e,f) Representative H&E staining of the cardiac sections (the top panel; Scale bar: 1 mm) and (the bottom panel; Scale bar: 100 µm) from each indicated group; f) the quantified data from the experiments shown in (e) demonstrating the inflammation areas (%; estimated by ImageJ Software) under each indicated condition (*n* = 6 for each group). g,h) Representative images of Sirius Red staining (upper panel) and Masson staining (lower panel) of cardiac sections from each indicated group; h) the quantified data from the experiments shown in (g) representing cardiac fibrosis areas (%; Masson staining analyzed by ImageJ Software) under the different experimental conditions (*n* = 6 for each group; Scale bar: 100 µm). i–n) Representative immunostaining demonstrating infiltration of neutrophils (Ly6G positive) (i), macrophages (F4/80 positive) (k), and *T*‐cells (CD3 positive) (m) in cardiac sections generated from each experimental group (scale bars equal 50 µm). Quantification data showing the absolute number of neutrophils j), macrophages l), and *T*‐cells (n) per field (*n* = 6 for each group; determined by ImageJ software). o,p) The relative gene expression of TNF‐α o), Il‐1α p), Il‐1β q), and IFN‐γ r) was analyzed by qPCR in mouse hearts from each experimental group (*n* = 5–6 for each group). Data are presented as the mean ± SEM. Statistics: one‐way ANOVA, followed by Tukey's post hoc multiple comparisons test was used for analyzing (b–d), (f,h,j,l,n,o‐r). The statistics were performed between indicated groups; ^**^, ^##^ and ^&&^ indicate *p <* 0.01.

To confirm this hypothesis, the EAM mice‐treated with either the anti‐IgG antibody or anti‐CTLA‐4 m2a antibody were received SB225002, a Cxcr2‐specific antagonist or a Cxcl1 neutralizing antibody, as depicted in Figure S1b (Supporting Information). Our data showed that anti‐CTLA‐4 m2a antibody‐induced cardiac dysfunction, increase in the serum cTnI levels, cardiac inflammation, and cardiac fibrosis in EAM mice were rescued by SB225002 or Cxcl1 neutralizing antibody in EAM mice (**Figure** **6**a–h). The data further demonstrated that CTLA‐4 m2a antibody‐induced increase in the infiltration of neutrophils (Ly6G^+^), macrophages (F4/80^+^), and T cells (CD3^+^) in the cardiac tissue of EAM mice were dramatically reduced by SB225002 or Cxcl1 neutralizing antibody (Figure 6i–n). Moreover, anti‐CTLA‐4 m2a antibody‐mediated increase in the expression levels of Tnf‐α, Il‐1α, Il‐1β, and INF‐γ in the EAM mouse cardiac tissues was significantly ameliorated by SB225002 or Cxcl1 neutralizing antibody (Figure 6o–r). These data are strongly supportive of the notion that strengthened Cxcl1‐Cxcr2 chemotaxis plays a critical role in stimulating infiltration of neutrophils in the hearts of anti‐CTLA‐4 m2a antibody‐treated EAM mice.

### 2.6 Anti‐CTLA‐4 m2a Antibody Alters Spatiotemporal Profiles of Immune Cells in the MZs of EAM Mice

**Figure 7:**
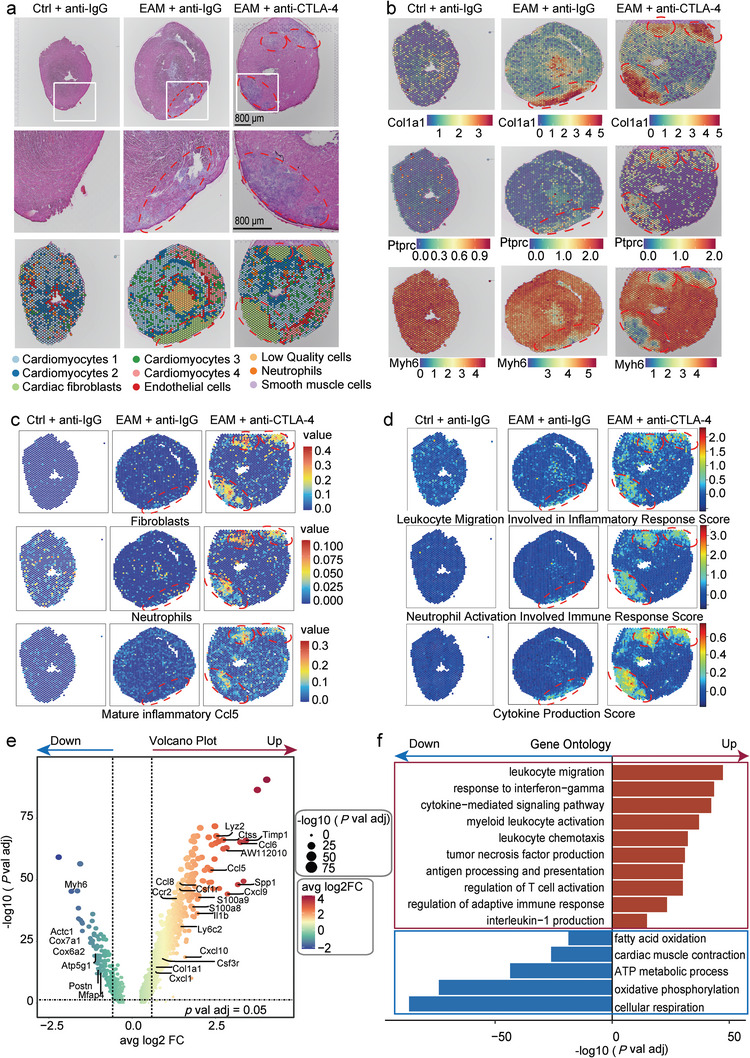
ST data revealed the dominant role of the Ccl5‐neutrophil subpopulation in the anti‐CTLA4 m2a antibody‐induced progression of myocarditis in EAM mice. a) H&E staining of cardiac tissue sections (the upper panel), the zoomed‐in views from where indicated by the white squares (the middle panel), and mapping with unbiased clustering of ST spots (Figure S8a, Supporting Information) were perfectly overlaid with the H&E images (the bottom panel) in each indicated experimental group. The cardiac MZs are circled by the dashed red lines. Scale bar: 800 µm. b) Spatial feature plots showing the relative expression levels of the marker genes Col1a1 (cardiac fibroblasts), Ptprc (immune cells), and Myh6 (cardiomyocytes) in the cardiac sections of each indicated experimental group. The colored bars shown on the bottom of each panel, coding from the blue to the red, indicate the relative gene expression levels from low to high. c) SPOTlight mapping on Visium ST from the cardiac sections demonstrating that localization of cardiac fibroblasts (upper panel), neutrophils (middle panel), and Ccl5‐neutrophil subpopulation (lower panel) within MZs was dramatically increased by anti‐CTLA‐4 m2a antibody in EAM mice. The colored bars, shown on the right of each panel, indicate the relative abundance of cardiac fibroblasts, neutrophils, and CCl5‐neutrophil subpopulation from low (the blue) to high (the red) (circled by the dashed red lines). d) ST maps showing that calculated GO terms scores for three interested proinflammatory cytokines signaling were dramatically enriched by anti‐CTLA‐4 m2a antibody in the MZs of EAM mice (circled by the dashed red lines). The colored bars shown on the right of each panel, coding from the blue to the red, indicate the signaling activity scores from low to high. e) Volcano plot showing DEGs in the MZs between anti‐IgG antibody‐ and anti‐CTLA‐4 m2a antibody‐treated EAM mouse hearts; the black dashed lines show the thresholds for significantly enriched genes. The depicted blue or red solid line with arrow head shown on the top respectively indicating that the expression of genes was decreased or increased by anti‐CTLA‐4 m2a antibody in the MZs compared with those in anti‐IgG antibody treated‐EAM mice. Genes with a false discovery rate adjusted *p‐*value < 0.05 and average log2FC > 0.5 were considered significantly regulated by the anti‐CTLA‐4 m2a antibody. f) GO analysis showing that the interest GO terms for genes enriched in the MZs were differed between anti‐IgG antibody‐ and anti‐CTLA‐4 m2a antibody‐treated EAM mice; fatty acid oxidation, cardiac muscle contraction, ATP metabolic process, oxidative phosphorylation, and cellular respiration signaling were significantly decreased by anti‐CTLA‐4 m2a antibody in the MZs of anti‐IgG antibody treated‐EAM mice (the blue solid rectangular bars); in contrast, leukocyte migration, response to interferon‐gamma, cytokine‐mediated signaling pathway, myeloid leukocyte activation, leukocyte chemotaxis, tumor necrosis factor production, antigen processing, and presentation, regulation of *T*‐cell activation, regulation of adaptive immune response and interleukin‐1 production were significantly increased by anti‐CTLA‐4 m2a antibody in the MZs of anti‐IgG antibody treated‐EAM mice (the red solid rectangular bars).

To explore the detailed spatial cellular features within the MZs, we performed spatial transcriptomics (ST) sequencing in the cardiac sections. The number and the area of MZs were increased by anti‐CTLA‐4 m2a antibody compared with those of anti‐IgG antibody‐treated EAM mouse hearts (**Figure** **7**a; highlighted by the dashed red circles; zoomed‐in images shown in the middle panel were from where indicated by the white rectangular squares). UMAP analysis revealed nine cell clusters (as reflected by the marker gene specific to each cluster) (Figure S8a, Supporting Information) with adequate quality (Figure S8b, Supporting Information). By projecting all ST clusters onto cardiac sections, we identified MZs (cardiac fibroblasts cluster) that were perfectly matched with the inflammatory areas determined by H&E staining. The data showed that cardiomyocytes cluster 1 and cardiomyocytes cluster 2 were localized to the border zone (BZ) and remote zone (RZ), respectively. In contrast, cardiac fibroblasts cluster localized to the MZs of cardiac tissue in both anti‐IgG antibody‐ and anti‐CTLA‐4 m2a antibody‐treated EAM mice (Figure 7a; highlighted by the dashed red circles). Moreover, anti‐IgG antibody‐induced increase in cardiac fibroblast marker gene Col1a1 and immune cell marker gene Ptprc were further increased by anti‐CTLA‐4 m2a antibody in the MZs of EAM mice; whereas anti‐IgG antibody‐induced decrease in cardiomyocyte marker gene Myh6 was further decreased by anti‐CTLA‐4 m2a antibody in the MZs of EAM mice (Figure 7b; highlighted by the dashed red circles). These data suggest that anti‐CTLA‐4 m2a antibody led to the further increased infiltration of immune cells, cardiac fibrosis, and loss of cardiomyocytes in the MZs of EAM mice.

To further depict the spatial features of the major cell types in the MZs, we mapped the scRNA data to the ST data using SPOTlight, as previously described.^[^ ^16^ ^]^ Our interrogated spatial data revealed that cardiac fibroblasts (Figure 7c; the upper panel highlighted by the dashed red circles) and neutrophils (Figure 7c; the middle panel highlighted by the dashed red circles) were exclusively localized within the MZs. Among the neutrophil subpopulations, Ccl5‐neutrophil was the major infiltrated subpopulation in the MZs (Figure 7c; the bottom panel highlighted by the dashed red circles and Figure S8c, Supporting Information). We calculated gene module scores for genes associated with ontology terms, including leukocyte migration involved in the inflammatory response, neutrophil activation involved in the immune response and cytokine production involved in immune response signaling. The data demonstrated that these pathways were enriched by anti‐CTLA‐4 m2a antibody in the MZs of EAM mice (Figure 7d; highlighted by the dashed red circles).

We also compared DEGs and performed GO enrichment analyses in the MZs between anti‐IgG antibody‐ and anti‐CTLA‐4 m2a antibody‐treated EAM mice. The data showed that the markers of infiltrated neutrophil (Lyz2, Csf1r Csf3r, S100a8, and S100a9), cardiac fibrosis (Col1a1), inflammation (Ly6c2 and IL‐1β), chemokine (Cxcl1, Cxcl9, Cxcl10, Ccl5, and Ccl8) and myocarditis (Timp1, AW112010, and Ctss) were significantly increased by anti‐CTLA‐4 m2a antibody (Figure 7e; highlighted by the dashed red circle and Figure S9, Supporting Information). GO term enrichment analysis showed that the proinflammatory signaling pathways in the MZs were significantly increased by anti‐CTLA‐4 m2a antibody in EAM mice (Figure 7f; highlighted by the red rectangular square); whereas the cardiac metabolic related signaling in the MZs was significantly decreased by anti‐CTLA‐4 m2a antibody in EAM mice (Figure 7f; highlighted by the blue rectangular square). ST data further revealed that proinflammatory signaling pathways were enriched in the MZs, as reflected by immune/cytokine scores (Figure S10, Supporting Information; highlighted by the dashed red circles). These results together suggest that the Ccl5‐neutrophil subpopulation plays a central role in stimulating anti‐CTLA4 m2a antibody‐induced progression of myocarditis and cardiac dysfunction in EAM mice.

### 2.7 Depleting Neutrophils Attenuates Anti‐CTLA‐4 m2a Antibody‐Induced Cardiac Injury in EAM Mice

**Figure 8:**
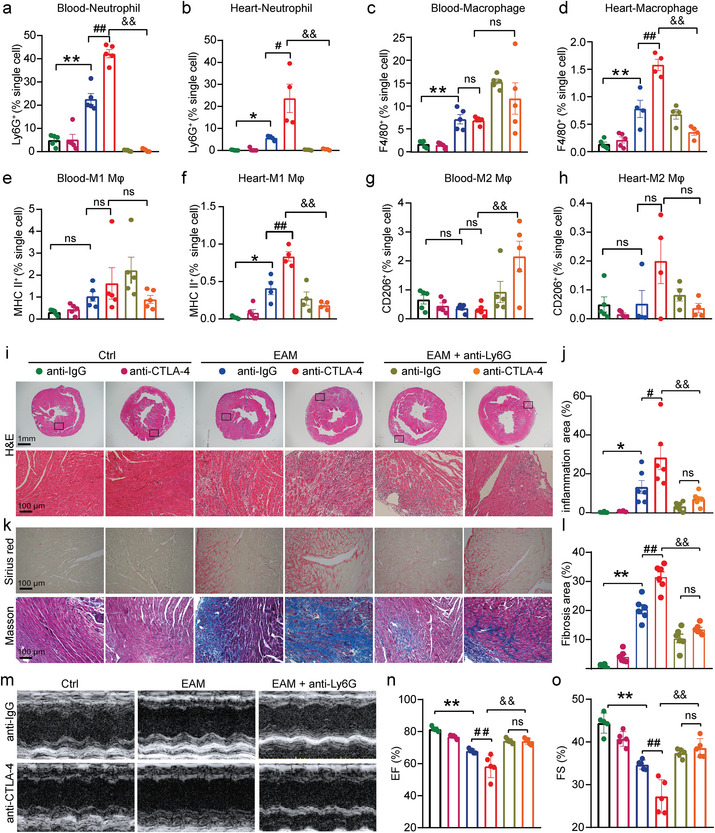
Depletion of neutrophils mitigates anti‐CTLA‐4 m2a antibody‐induced cardiac inflammation, fibrosis, and dysfunction in EAM mice. a–h) Quantification analyses of flow cytometry, obtained from the peripheral blood of each indicated experimental group, demonstrating the percentage of CD45^+^CD11b^+^Ly6G^+^F4/80^−^ neutrophils a), CD45^+^CD11b^+^ Ly6G^−^F4/80^+^ macrophages c), CD45^+^CD11b^+^F4/80^+^MHCII^+^ M1 Mφ e), and CD45^+^CD11b^+^ F4/80^+^CD206^+^ M2 Mφ g) to total cells (*n* = 5 for each group). Quantification analyses of flow cytometry, generated from cardiac tissues of each indicated experimental group, demonstrating the percentage of CD45^+^CD11b^+^Ly6G^+^F4/80^−^ neutrophils b), CD45^+^CD11b^+^ Ly6G^−^F4/80^+^ macrophages d), CD45^+^CD11b^+^F4/80^+^MHCII^+^ M1 Mφ f), CD45^+^CD11b^+^ F4/80^+^CD206^+^ M2 Mφ h) to total cells (*n* = 5 for control groups; *n* = 4 for EAM or EAM + anti‐Ly6G antibody groups). i,j) Representative H&E staining of the cardiac sections (the top panel; Scale bar: 1 mm) and (the bottom panel; Scale bar: 100 µm) from each indicated group; j) the quantified data from the experiments shown in (i) demonstrating the inflammation areas (%; estimated by ImageJ Software) under each indicated conditions (*n* = 6 for each group). k,l) Representative images of Masson staining (upper panel) and Sirius Red staining (lower panel) of cardiac sections from each indicated group; l) the quantified data from the experiments shown in (k) representing cardiac fibrosis areas (%; Masson staining analyzed by ImageJ Software) under the different experimental conditions (*n* = 6 for each group; Scale bar: 100 µm). m–o) Representative M‐mode view of echocardiography obtained from each indicated experimental group m); summarized mean data of ejection fraction (EF) n) and fractional shortening (FS) o) from each indicated group (*n* = 5 for each group). Data are presented as the mean ± SEM. Statistics: one‐way ANOVA, followed by Tukey's post hoc multiple comparisons test was used for analyzing (a–h,j,l,n,o). The statistics were performed between indicated groups; ns represents *p* > 0.05; ^*^ and ^#^ indicate *p <* 0.05; ^**^, ^##^ and ^&&^ indicate *p <* 0.01.

To explore the potential role of neutrophils in anti‐CTLA‐4 m2a antibody‐associated cardiac injury in EAM mice, anti‐CTLA‐4 m2a and anti‐Ly6G antibodies were concurrently administered to mice on the 14th day post initial immunization, as depicted in Figure S1c (Supporting Information). Flow cytometry (the gating strategies depicted in Figure S11, Supporting Information) demonstrated that neither anti‐IgG antibody nor anti‐CTLA‐4 m2a antibody alone altered the ratios of neutrophil and macrophage in both peripheral blood and cardiac tissues of control mice (**Figure** **8**a–d). The ratios of neutrophil were significantly increased in both peripheral blood and cardiac tissues of anti‐IgG antibody or anti‐CTLA‐4 m2a antibody‐treated EAM mice, with a much higher extent in anti‐CTLA‐4 m2a antibody injected group. The increased ratios in both anti‐IgG antibody‐ and anti‐CTLA‐4 m2a antibody‐treated EAM mice were diminished by anti‐Ly6G antibody, a neutrophil neutralizing agent (Figure 8a,b).

Anti‐CTLA‐4 m2a antibody did not affect the ratio of macrophages in the peripheral blood of EAM mice (Figure 8c); however, the elevated ratio of macrophage, seen in the cardiac tissues of anti‐IgG antibody‐treated EAM mice, was further increased by anti‐CTLA‐4 m2a antibody and was dramatically decreased by anti‐Ly6G antibody (Figure 8d). Interestingly, anti‐Ly6G antibody did not significantly affect the ratio of macrophage in the peripheral blood of both anti‐IgG and anti‐CTLA‐4 m2a antibodies‐treated EAM mice (Figure 8c). Moreover, EAM‐induced elevation of the M1 Mφ ratio in the cardiac tissues was further increased by anti‐CTLA‐4 m2a antibody, which was dramatically attenuated by anti‐Ly6G antibody (Figure 8f). The ratios of M1 Mφ and M2 Mφ in the peripheral blood (Figure 8e,g) and the ratio of M2 Mφ in the cardiac tissue (Figure 8h) were not significantly altered by either the anti‐IgG antibody or anti‐CTLA‐4 m2a antibody in EAM mouse hearts. Moreover, anti‐IgG antibody or anti‐CTLA‐4 m2a antibody alone did not cause any notable cardiac inflammation, fibrosis, or dysfunction in control mice. The increased inflammatory and fibrosis areas, and cardiac dysfunction seen in anti‐IgG antibody‐treated EAM mice were further worsened by anti‐CTLA‐4 m2a antibody and were dramatically attenuated by anti‐Ly6G antibody (Figure 8i‐o).

## 3 Discussion

The major new findings of this study are: 1) cardiac fibroblast‐derived Cxcl1 drives neutrophil infiltration via Cxcr2 to the MZs in anti‐CTLA‐4 m2a antibody‐treated EAM mice; 2) Ccl5‐neutrophil subpopulation plays a critical role in exacerbating inflammation, cardiac fibrosis and dysfunction, through elevating proinflammatory cytokine/chemokine‐mediated macrophage polarization toward M1 Mφ in the MZs of anti‐CTLA‐4 m2a antibody‐treated EAM mice; 3) neutralizing neutrophils, Cxcl1 or applying Cxcr2 antagonist ameliorates anti‐CTLA‐4 m2a antibody‐induced cardiac injury. Our data provide the rationale for preventing ICIs‐associated myocarditis.

Patients, with preexisting coronary syndrome or heart failure and aged over 75 years old, are associated with an increased risk of myocarditis.^[^ ^2^ ^]^ Recent studies showed that patients with AD have a two‐fold higher risk for suffering cardiovascular events including ICIs‐associated myocarditis.^[^ ^3^ ^]^ Indeed, α‐myosin‐expanded TCRs in circulatory *T*‐cells of ICIs‐induced myocarditis patients were significantly increased.^[^ ^9^ ^]^ These findings suggest that preexisting conditions and α‐myosin may increase the susceptibility to ICIs‐associated myocarditis. Therefore, we investigated whether anti‐CTLA‐4 m2a antibody could exacerbate myocarditis using the EAM model. Among the nine identified cell clusters in our experimental model, the neutrophil and macrophage were the two largest fractions that infiltrated within the MZs of EAM mice. This is consistent with a recent study that showed that innate immune cells including macrophages and neutrophils represented as the greatest proportion of the total immune cells in EAM mice.^[^ ^17^ ^]^ Consistent with immunohistochemistry data, scRNA‐seq data revealed that the amount of infiltrated neutrophils and T cells within the MZs was significantly increased by anti‐CTLA‐4 m2a antibody in EAM mouse hearts. However, the relative fraction of infiltrated macrophage was significantly decreased by anti‐CTLA‐4 m2a antibody in EAM mouse hearts, suggesting that the altered landscape of immune cells by anti‐CTLA‐4 m2a antibody is rather complex. Therefore, we further deciphered the underlying mechanisms. scRNA‐seq data showed that among the nine identified cell types, neutrophils appeared to exhibit the highest inflammatory response scores. This notion was confirmed by the quantitative PCR (qPCR) experiments, where EAM‐induced increase in proinflammatory cytokines production in the cardiac tissues, including Tnf‐α, Il‐1α, Il‐1β, and INF‐γ, were further increased by anti‐CTLA‐4 m2a antibody.

*T*‐cell‐specific CTLA‐4 condition knockout (KO) resulted in inflammation in multi‐organs including myocarditis and led to the preferential expansion of frequency and absolute number of CD4^+^Foxp3^+^ Treg cells, but not CD8^+^ *T*‐Cells, in spleen and lymph nodes.^[^ ^5^ ^]^ Surprisingly, inducible CTLA‐4 KO in *T*‐cells of adult mice also caused systemic inflammatory phenotypes without signs of myocarditis.^[^ ^5^ ^]^ Similar to the cardiac phenotype of inducible *T*‐cell CTLA‐4 KO, we showed that injecting anti‐CTLA‐4 m2a antibody alone did not cause any remarkable immune cell infiltration and the signs of cardiac injury in control mice. In contrast, the proportion of infiltrated CD4^+^ Treg cells was significantly elevated and the fraction of infiltrated Th17 like cells was significantly decreased by anti‐CTLA‐4 m2a in EAM mouse hearts. It was considered that anti‐CTLA‐4 m2a antibody depletes Foxp3^+^ Treg cells in several tumor‐bearing mice,^[^ ^18^ ^]^ however, the density of Foxp3^+^ Treg cells within the microenvironment of the primary prostate tumor was significantly increased by anti‐CTLA‐4 m2a antibody.^[^ ^19^ ^]^ These results together suggest that the regulation of Treg cells, the primary target cell of anti‐CTLA‐4 m2a antibody, may vary between the different experimental models or clinical conditions and may also depend upon the Fc fragment of anti‐CTLA‐4 m2a antibody.^[^ ^20^ ^]^ Nevertheless, our experimental evidence suggests that anti‐CTLA‐4 m2a antibody‐mediated alteration of infiltrated *T*‐cell subpopulation may not be the direct cause for worsening the cardiac phenotypes in EAM mice.

Neutrophils act as the first responders to sites of injury to coordinate the initial proinflammatory response.^[^ ^21^ ^]^ For instance, mobilization and infiltration of neutrophils were earlier than any other immune cell in coxsackievirus B3 (CVB3)‐induced myocarditis.^[^ ^22^ ^]^ Depletion of neutrophils significantly reduced myocardial infarction size in ischemia/reperfusion injury mouse model^[^ ^23^ ^]^ and could ameliorate angiotensin II (Ang II)‐induced pathological cardiac hypertrophy and heart failure.^[^ ^24^ ^]^ The enhanced cardiac inflammation appeared to be tightly associated with severe neutrophil infiltration in the hearts of PD‐1 deficient EAM mice.^[^ ^25^ ^]^ Moreover, in a fibrosarcoma (MCA‐205) tumor‐bearing mouse model, anti‐CTLA‐4 m2a antibody led to an accumulation of neutrophils and immune reorganization at the early stage (8 days after treatment).^[^ ^20c^ ^]^ Our data revealed that the increased infiltration of neutrophils and neutrophil‐associated inflammatory cytokines appeared to be very important in anti‐CTLA‐4 m2a antibody‐induced cardiac injury in EAM mice. Therefore, we identified which subpopulation of neutrophils plays the most dominant role during the dynamic progress of inflammatory responses in anti‐IgG antibody‐ and anti‐CTLA‐4 m2a antibody‐treated EAM mouse hearts. We suggest that the infiltrated proportion and, differentiation of neutrophils and neutrophil‐associated transcriptional signatures are distinct between anti‐IgG antibody‐ and anti‐CTLA‐4 m2a antibody‐treated EAM mice. Our scRNA‐seq data showed the proportion of inflammatory mature Ccl5‐neutrophil subpopulation was dramatically increased by the application of anti‐CTLA‐4 m2a‐antibody in EAM mice. In anti‐IgG antibody‐treated EAM mice, immature Ltf‐neutrophil subpopulation differentiated toward Cell fate 1 (Ifitm2‐ and Ccl5‐neutrophil subpopulation), with higher activity in the cytokine‐meditated signaling pathway, leukocyte cell‐cell adhesion, and neutrophil migration. In contrast, anti‐CTLA4 m2a antibody led differentiation of immature Ltf‐neutrophil subpopulation toward Cell fate 2 (Ccl5‐neutrophil subpopulation), with the significantly enhanced transcriptional signatures of the immune and inflammatory activities including positive regulation of inflammatory response, leukocyte chemotaxis, response to interferon‐gamma. Moreover, we revealed that the mRNA expression levels of Ccl5‐neutrophil subpopulation‐associated chemokines (Ccl4, Cxcl10, and Cxcl9) and proinflammatory cytokines (Il‐1α and Tnf‐α) were significantly increased by anti‐CTLA‐4 m2a antibody in the cardiac tissue of EAM mice. More importantly, the results described above, combined with the data obtained from ST analyses, demonstrated that the Ccl5‐neutrophil subpopulation and its‐associated proinflammatory cytokines and chemokines were dramatically enriched by anti‐CTLA‐4 m2a antibody in the MZs of EAM mice, suggesting the critical role of increased infiltration of Ccl5‐neutrophil subpopulation for mediating anti‐CTLA4 m2a antibody‐induced cardiac injury in EAM mice. However, this notion is limited by lacking direct experimental evidence as there is no effective tool available to specifically deplete Ccl5‐neutrophil subpopulation.

We next deciphered what factor triggers neutrophil infiltration to the MZs. It is well known that proinflammatory chemokines mediate the trafficking and recruitment of leucocytes to the sites of inflammation. Cxcl1‐Cxcr2 axis‐mediated infiltration neutrophils and monocytes/macrophages into the heart and vascular wall plays a central role in the pathogenesis of Ang II‐induced cardiac hypertrophy and hypertension.^[^ ^27^ ^]^ Emerging evidence suggests that activated cardiac fibroblasts could secrete different cytokines and chemokines in response to pathological stimuli, such as transverse aortic constriction and myocardial infarction models of heart failure.^[^ ^28^ ^]^ We revealed for the first time that chemotaxis between EAM‐induced increase in cardiac fibroblasts‐derived Cxcl1 and Cxcr2 of neutrophils promoted the recruitment of neutrophils into the heart of EAM mice. The mRNA levels of Cxcl1 were significantly increased by anti‐CTLA‐4 m2a antibody in the cardiac tissue of EAM mice, suggesting that anti‐CTLA‐4 m2a antibody may further elevate Cxcl1 production by cardiac fibroblasts. Moreover, neutrophils and Cxcl1 were highly co‐localized by anti‐CTLA‐4 m2a antibody within the MZs of EAM mice, suggesting that anti‐CTLA‐4 m2a antibody led to positive feedback of Cxcl1‐Cxcr2 axis to further promote infiltration of neutrophils. This was confirmed by the rescue experiments, where anti‐CTLA‐4 m2a antibody‐induced increase in neutrophil infiltration in the MZs, enhanced cardiac inflammatory responses, cardiac fibrosis, and dysfunction in EAM mice were dramatically ameliorated by both SB225002 and Cxcl1 neutralizing antibody. In addition, our data also suggest that the expression of Cxcl9 and Cxcl10, two chemokines highly related to CD8^+^ T cell infiltration in human solid tumors,^[^ ^29^ ^]^ were increased by anti‐CTLA‐4 m2a antibody in the MZs of EAM mice. However, the number of infiltrated CD8^+^ effector *T*‐cells was not significantly altered by anti‐CTLA‐4 m2a antibody in the MZs of EAM mice in our hands; the function of CD8^+^ effector *T*‐cells infiltrated in the cardiac tissues of EAM mice appeared to be inhibited by anti‐CTLA‐4 m2a antibody. These phenomena are probably attributed to the increased Treg cells in anti‐CTLA‐4 m2a antibody‐treated of EAM mouse hearts.

Our immunohistochemistry data showed that anti‐CTLA‐4 m2a antibody significantly increased the absolute number of macrophage infiltration in the hearts of EAM mice. Surprisingly, scRNA‐seq data suggested that the absolute number of macrophages, from the sorted CD45^+^ leukocytes of EAM mouse hearts, appeared to be decreased by anti‐CTLA‐4 m2a antibody. This contradictory result compared with the immunohistochemistry findings may arise from the differences between the two experimental methodologies. The absolute number of infiltrated macrophages in the MZs were directly counted in the immunohistochemistry analyses, allowing for a straightforward visual quantification of the cells albeit varying lesion severity in the selected areas may also impact overall counts. In contrast, the samples for scRNA‐seq analyses were generated from the heart tissues and were loaded nearly equal amounts of CD45^+^ leukocytes. This approach may potentially reduce the observed absolute number of all cell types, since the overall number of CD45^+^ leukocytes was significantly increased by anti‐CTLA‐4 m2a antibody in EAM mouse hearts. Among the identified CD45^+^ leukocytes, the absolute number and proportion of neutrophils were drastically increased by anti‐CTLA‐4 m2a antibody in the EAM mouse heart tissues. This shift in cell composition may affect the overall cell type representation in scRNA‐seq data, thereby contributing to the observed lower macrophage counts. More importantly, scRNA‐seq data showed that anti‐CTLA4 m2a antibody significantly increased the proportion of M1 Mφ subpopulation and decreased the fraction of M2 Mφ subpopulation in the hearts of EAM mice. A recent study also showed that the infiltration of inflammatory macrophages was significantly increased in the heart of an ICIs‐associated myocarditis mouse model (Ctla‐4^+/−^Pdcd1^−/−^ mice).^[^ ^30^ ^]^ The question remained to be addressed whether the increased proportion of M1 Mφ subpopulation is attributable to anti‐CTLA4 m2a antibody‐mediated infiltration of Ccl5‐neutrophil subpopulation, albeit Ccl5‐neutrophil subpopulation‐mediated interferon gamma signaling and response to Tnf was significantly increased in the MZs. The other evidence from flow cytometry experiments also suggests that infiltration of was the earlier event than M1 Mφ polarization because the anti‐Ly6G antibody significantly reversed the anti‐CTLA‐4 m2a antibody‐induced increase in M1 Mφ in the hearts of EAM mice.

Integrated scRNA‐seq and ST data were recently used to study cardiovascular diseases;^[^ ^31^ ^]^ however, to our knowledge, the cellular heterogeneity with spatial information of the MZs has not been characterized in ICIs‐associated myocarditis. In the current study, we combined scRNA‐seq and ST data to obtain spatiotemporal expression profiles of the MZs at the single‐cell level. Differential expression analysis showed that the much lower levels of Cox7a1, Cox6a2, Actc1, and Atp5g1 were expressed within MZs, with downregulated genes involved in oxidative phosphorylation, cardiac muscle contraction, fatty acid metabolic process, and ATP metabolic process in anti‐CTLA‐4 m2a antibody‐treated EAM mice. Consistent with the data obtained from viral myocarditis,^[^ ^31^ ^]^ we found that myocardial injury‐related genes including Timp1, AW112010, and Ctss were significantly increased by anti‐CTLA4 m2a antibody in the MZs of EAM mice.

In summary, we conclude that the mechanisms of anti‐CTLA4 m2a antibody‐induced cardiac injury of EAM mice are involved in alterations of immune‐, proinflammatory cytokines/chemokines‐mediated cardiac metabolic dysfunction, loss of cardiomyocytes, and fibrosis, in which Ccl5‐neutrophil subpopulation appears to act as a critical role. Our finding may provide an attractive therapeutic strategy for preventing and curing ICIs‐associated myocarditis.

## 4 Experimental Section

A detailed Materials and Methods section is available in the online‐only Data Supplement.

## Conflict of Interest

The authors declare no conflict of interest.

## Author Contributions

M.‐M.W., Y.‐C.Y., Y.‐X.C., and S.J. contributed equally to this work. Zhi‐Ren Zhang designed the study and revised the manuscript; Z.‐W.P. provided critical insights to the study; M.‐M.W., Y.‐C.Y., Y.‐X.C. and S.J. performed most of the experiments and draft the manuscript; H.X., Y.S., X.B., Z.H., D.Z., M.Y., C.M., J.‐J.M., C.‐J.Y., C.L., L.‐L.T., Q.S.W., Q.S. performed some of the experiments; M.‐M.W., Y.‐C.Y., X.‐Y.J. and Q.‐H.J. analyzed data and prepared Figures. All authors have read and approved the final version of the manuscript.

## Supporting information

Supporting Information

Supporting Information

## Data Availability

The raw data have been deposited in the GSA (Genome Sequence Archive) databases of the National Genomics Data Center (NGDC, https://bigd.big.ac.cn/ under the BioProject accession code: PRJCA027500. The data that support the findings of this study are available from the corresponding author upon reasonable request.
