# Supplementary material for: Anti‐CTLA‐4 m2a Antibody Exacerbates Cardiac Injury in Experimental Autoimmune Myocarditis Mice By Promoting Ccl5‐Neutrophil Infiltration
Source: Adv Sci (Weinh). 2024 Jul 8;11(34):2400486. doi: 10.1002/advs.202400486 (PMC11425905; doi:10.1002/advs.202400486)
Supplement: Supplementary file 1 — Supporting Information [file ADVS-11-2400486-s002.docx]

**Supporting Information**

**Anti-CTLA-4 m2a Antibody Exacerbates Cardiac Injury in Experimental Autoimmune Myocarditis Mice** **By Promoting Ccl5-Neutrophils Infiltration**

**Authors:** Ming-Ming Wu^1, 2, 3, #^, Yan-Chao Yang^1, 2, #^, Yong-Xu Cai^2, #^, Shuai Jiang^2, #^, Han Xiao^1^, Chang Miao^1^, Xi-Yun Jin^4^, Yu Sun^1^, Xin Bi^1^, Zi Hong^1^, Di Zhu^1^, Miao Yu^1,2^, Jian-Jun Mao^1, 2^, Chang-Jiang Yu^2^, Chen Liang^1, 2^, Liang-Liang Tang^2^, Qiu-Shi Wang^1^, Qun Shao^2^, Qing-Hua Jiang^4^, Zhen-Wei Pan ^3,5^ and Zhi-Ren Zhang^1, 2, 3^*

^1^Departments of Cardiology and Critical Care Medicine, the First Affiliated Hospital of Harbin Medical University (HMU), NHC Key Laboratory of Cell Transplantation, Key Laboratories of Education Ministry for Myocardial Ischemia Mechanism and Treatment, National Key Laboratory of Frigid Zone Cardiovascular Zone, Harbin, 150001, China

^2^Departments of Cardiology and Pharmacy, HMU Cancer Hospital, Institute of Metabolic Disease, Heilongjiang Academy of Medical Science, Heilongjiang key laboratory for Metabolic disorder and cancer related cardiovascular diseases, Harbin, 150081, China

^3^State Key Laboratory of Frigid Zone Cardiovascular Diseases (SKLFZCD), HMU, Harbin, 150081, China

^4^School of Interdisciplinary Medicine and Engineering, HMU, Harbin, 150081, China

^5^Department of Pharmacology (State-Province Key Laboratories of Biomedicine-Pharmaceutics of China, Key Laboratory of Cardiovascular Medicine Research, Ministry of Education), HMU, Harbin, 150081, China

#These authors contributed equally

*To whom correspondence should be addressed: Zhi-Ren Zhang, M.D., Ph.D.

Departments of Cardiology and Critical Care Medicine, NHC Key Laboratory of Cell Transplantation, The First Affiliated Hospital of HMU and HMU Cancer Hospital, SKLFZCD; No. 23 Youzheng Street, Harbin, 150001, China

Tel: +86-451-5360 8395

**This PDF file includes:**

Supplemental Methods

Supplemental Figures. S1 to S11

Tables S1

**Other Supplemental Materials for this manuscript include the following:**

Excel Data S1

**Supplemental Methods**

**Animals**

Male BALB/c mice (6 weeks of age) were purchased from Beijing Vital River Laboratory Animal Technology (Beijing, China). All animal experimental procedures were performed according to the guidelines from ARRIVE and the U.S. NIH. ^[1]^ In addition, all experimental protocols were approved by the Ethical Committee of Harbin Medical University for Animal Research (KY2022-15). Mice were housed in a climate-controlled light-regulated space with a 12/12 light cycle and received food and water *ad libitum* at the Animal Center of the Second Affiliated Hospital of Harbin Medical University.

To generate an EAM mouse model, the mice were developed by injecting murine α-cardiac myosin heavy chain (α-myosin) fragment (α614-629, Ac-RSLKLMATLF STYASADR-OH; GL Biochem, Shanghai, China) as described previously. ^[2]^ Briefly, the mice were subcutaneously injected with 200 μg α-myosin emulsified with complete Freund adjuvant (Sigma, F5881; 1:1, wt wt^-1^) on days 0 and 7. On day 0 (initial immunization), the mice were also intraperitoneally injected with 500 ng of pertussis toxin (Absin abs42024900). Mice, administered with normal saline (NS) mixed with an equal volume of Freund's complete adjuvant and 500 ng of pertussis toxin, were used as the control group. Anti-CTLA-4 m2a antibody (10 mg kg^-1^, Bioxcell, BE0032) or anti-IgG antibody (10 mg kg^-1^, Bioxcell, BE0089) was intraperitoneally injected per mouse on days 14, 16, 18, and 20 post initial immunization, as presented in Figure S1a. According to the doses reported by the previous clinical trials and the experiments conducted in mice ^[3]^, we used a clinical relevant dose 10 mg kg^-1^ anti-mouse CTLA-4 m2a antibody in this study. For rescue experiments, SB225002 (10 mg kg^-1^, Selleck Chemicals, #S7651), a Cxcr2 specific inhibitor was administered intraperitoneally on days 14 to 20 (daily), as previously reported; ^[4]^ the anti-Cxcl1 antibody (5 mg kg^-1^, Thermo-Fischer, #MA5-23745)^[5]^ or the anti-ly6G antibody (10 mg kg^-1^, Bioxcell, #BE0075) was administered intraperitoneally concurrently with anti-CTLA-4 m2a antibody on days 14, 16, 18, and 20 post initial immunization (depicted in Figure S1b, c). The blood and hearts were collected for further analysis on day 21 post the initial immunization.

**Echocardiography**

Echocardiography was performed using a Vevo2100 High-Resolution Imaging System (VisualSonics, Toronto, Canada) at the indicated times to evaluate mouse cardiac function, as previously reported. ^[6]^ After anesthetization with 2.5% avertin (0.02 mL g^-1^ of body weight), the mice were placed in a supine position. A 15-MHz transducer was applied to the left hemithorax. The heart was imaged in short-axis views with a depth setting of 2 cm. The cursor was positioned perpendicular to the interventricular septum and posterior wall of the left ventricular (LV) at the level of the mid‐papillary muscle, with tracing with a sweep speed of 50 mm/s for measurement of LV end‐systolic diameter (LVESD) and LV end‐diastolic diameter (LVEDD). The percentage of fractional shortening (FS) was calculated using the following formula: FS % = (LVEDD – LVESD)/LVEDD × 100%, and ejection fraction (EF) was calculated using the following formula: EF % = (LVEDV – LVESV)/LVEDV × 100%.

**Histopathology**

The hearts were harvested and perfused with 10 mL of PBS through the aortic arch puncture. Cardiac samples were fixed in 4% paraformaldehyde (PFA) for 48 hours and dehydrated with graded ethanol. The samples were embedded in paraffin, cut into slices (4 μm thick), dewaxed, and rehydrated. Samples were then separately stained with hematoxylin and eosin (H&E), periodic acid schiff (PAS) and Masson staining for cardiac inflammation and fibrosis. The histological changes were observed using a BX53 upright microscope (Olympus, Tokyo, Japan). Finally, quantitative analyses were performed by using a quantitative digital image analysis system (NIH ImageJ software, Bethesda, MD, USA). ^[7]^

For immunohistochemistry staining, cardiac sections were deparaffinized and hydrated by decreasing ethanol. To reveal antigens, the sections were incubated in 1 mM Tris solution (pH 9.0) supplemented with 0.5 mM EGTA and heated in a microwave oven for 5 min. Endogenous peroxidase was blocked by incubating with 3% H_2_O_2_ for 10 min at room temperature (RT; 22-24℃), followed by blocking with 5% bovine serum albumin (BSA) in PBS for 1 hour at RT. Sections were incubated with anti-Ly6G (Invitrogen, 14-5931-82, 1:200 dilution), anti-F4/80 (Invitrogen, 14-4801-82, 1:200 dilution) and anti-CD3 (Abcam, ab11089,1:200 dilution) at 4℃ overnight, followed by horseradish peroxide-conjugated rabbit anti-rat secondary antibody (Abcam, ab6734, 1:500 dilution) for 1 hour at RT. After washing with PBS, the sections were visualized by the HRP-DAB detection method, counterstained with hematoxylin, and dehydrated by ascending order of ethanol and xylene. Images were acquired using a BX53 upright microscope (Olympus, Tokyo, Japan).

The paraffin-embedded cardiac sections (4 μm thick) were used for immunofluorescence staining as pervious described. ^[8]^ The slides were boiled in EGTA antigen retrieval solution for 5 min for epitope retrieval. After blocking with 1% BSA for 1 hour at RT, the sections were incubated overnight at 4°C with Cxcl1 antibody (Abcam, ab86436,1:100 dilution) and vimentin antibody (Abcam, ab8978, 1:100 dilution) in PBS supplemented with 1% BSA. The sections were incubated with Alexa Cy3-conjugated donkey anti-rabbit (Jackson ImmunoResearch, 711-166-152, 1:1,000 dilution) or Alexa Fluor 488-conjugated goat anti-mouse (Invitrogen A11001, 1:1,000 dilution) for 1 hour. Co-immunofluorescent staining assays were performed to detect colocalization of the neutrophil and the cytokines (Il-1α, Il-1β and INF-γ). The mouse hearts were fixed with 4% PFA overnight followed by 18% sucrose 16 hours, then preserved in optimum cutting temperature compound (-80°C). The frozen cardiac tissue samples (4 μm-thick sections) were permeabilized with 0.2% Triton X-100 (PBST) for 10 min and blocked with 5% bovine serum albumin (BSA) PBST for 1 hour. The anti-Ly6G antibody (Invitrogen, 14-5931-82, 1:200 dilution) with anti-IL-1α antibody (Proteintech, 16765-1-AP, 1:100 dilution), anti-IL-1β antibody (Proteintech, 26048-1-AP, 1:100 dilution), or anti-IFN-γ polyclonal antibody (Invitrogen, PA5-95560, 1:300 dilution) were added in 1% BSA/PBST for overnight at 4°C. Then, the sections were washed in PBST and incubated with Alexa Fluor 488 conjugated donkey anti-rabbit (Jackson ImmunoResearch, 711-546-152, 1:800 dilution) with Cy3-conjugated donkey anti-rat (Jackson ImmunoResearch, 712-166-153, 1:800 dilution) for 1 hour at RT. For co-immunofluorescent staining of neutrophils with TNF-α, the anti-Ly6G antibody were added in 1% BSA/PBST for overnight at 4°C. On the following day, the sections were incubated with Cy3-conjugated donkey anti-rat (Jackson ImmunoResearch, 712-166-153, 1:800 dilution) and CoraLite® Plus 488-conjugated anti-TNF-α monoclonal antibody (Proteintech, CL488-60291, 1:100 dilution) for 1 hour at RT. Cell nuclei were counterstained with Hoechst 33342 (H1399, Invitrogen Carlsbad, CA, USA). All slides were imaged using a confocal microscope (Olympus, Fluoview1000, Japan). Identical acquisition settings were used for all images.

**Real-time** **quantitative PCR (qPCR) analysis**

Total RNA was extracted from mouse hearts using TRIzol reagent (Invitrogen, Carlsbad, CA, USA) and reverse transcribed into cDNA using the High-Capacity cDNA Reverse Transcription kit (Applied Biosystems, #4368813). Then qPCR was performed using the BIO-RAD CFX96 Real-Time System with SYBR Green qPCR Master Mix (B21203, Bimake, Shanghai, China). The relative expression levels of target genes were normalized to the endogenous housekeeping gene GAPDH using the ΔΔCt method. Results were expressed as a fold change relative to the control samples as the baseline. Primer sequences were synthesized by Sangon Biotech (Shanghai, China) and listed Supplementary Table 1.

**ELISA for quantification of Cxcl1 and cTnI**

The peripheral blood was obtained from each of the mice at the time of sacrifice. ELISA was performed to measure the levels of serum Cxcl1 (#H306-1) and cTnI (#H149-2-2) in each indicated experimental group using ELISA Kit (Nanjing Jiancheng Bioengineering Institute, Nanjing, China), according to the manufacturer’s recommendations. Standard curves were created using the CurveExpert 1.3 software program.

**Heart isolation and single-cell preparation**

The mouse hearts were harvested and perfused with PBS through aortic arch puncture after anesthetization. The cardiac tissues were enzymatically digested with Miltenyi tissue isolation kit 2 (Miltenyi Biotec, #130-110-203) according to the manufacturer's instructions. Briefly, five hearts from each group were minced and dissociated with enzyme solution enzyme mix 1 (2362.5 μL) and enzyme mix 2 (137.5 μL) for 15 min at 37℃ and vortexed with a GentleMacs Dissociator (Miltenyi Biotec, #130-095-937). After dissociation, the single-cell suspension was passed through a 40 μm filter and washed twice with PBS, and then the Debris Removal Solution (Miltenyi Biotec, 130-109-398) was added to remove cell debris. Red blood cell lysis solution (Miltenyi Biotec, #130-094-183) was added and incubated on ice for 2 min. The cells were resuspended in PBS, and the cell viability was greater than 85% as confirmed by trypan blue staining.

**Flow cytometry and fluorescence-activated cell sorting**

The peripheral blood samples were collected from the retro-orbital sinus after anesthesia and lysed using red blood cell lysis solution (Miltenyi Biotec, #130-094-183). Peripheral blood leukocytes and cardiac single cell suspensions were stained with anti-CD45 FITC (BioLegend, #103108, 1.2 µg mL^-1^), anti-CD11b APC (BioLegend, #101212, 0.6 µg mL^-1^), anti-Ly6G Alexa Fluor® 700 (BD Biosciences, # 561236, 0.6 µg mL^-1^), F4/80 BV421 (BioLegend, #123132, 1 µg mL^-1^), PerCP anti-mouse I-A/I-E Antibody (Biolegned, #107624, 0.5 µg mL^-1^), and PE anti-mouse CD206 (MMR) Antibody (Biolegned, #141706, 0.5 µg mL^-1^). Cells were gated on singlets, and leucocyte (CD45^+^ cells) subpopulations were defined as follows (Gating strategy represented in supplementary material online, Figure S10a, b): neutrophils (CD45^+^CD11b^+^F4/80^-^Ly6G^+^), macrophage (CD45^+^CD11b^+^Ly6G^-^F4/80^+^) M1 Mφ (CD45^+^CD11b^+^Ly6G^-^F4/80^+^MHCII^+^) and M2 Mφ (CD45^+^CD11b^+^Ly6G^-^F4/80^+^ CD206^+^). Flow cytometry data were analyzed using FlowJo (v.10.7.1, FlowJo, BD Biosciences).

Single-cell suspensions from mouse hearts were washed and resuspended in PBS. Cells were suspended in 500 μL staining buffer containing anti-CD45 FITC (BioLegend, #103108, 1.2 µg mL^-1^) at a dilution of 1:200 per 10^6^ cells, incubated at RT for 20 min in the dark, and then washed twice with PBS. The cells were stained with 7-AAD (559925, BD Biosciences, 1:20). Prepared single cells were sorted using a FACS Aria II cell sorter (BD Biosciences, CA, USA). Then, viable leukocytes (7-AAD^-^CD45^+^) were subjected to further scRNA-seq.

**Sequencing and analysis of scRNA-seq data**

Non-cardiomyocytes were respectively generated from anti-IgG antibody- and anti-CTLA-4 m2a antibody-treated EAM mouse hearts. The single cells were suspended in 0.04% BSA/PBS. The cell counts and viability were verified by TC20™ Automated Cell Counter (Thermo Fisher). The Chromium Single Cell VDJ 5' Reagent V2 Kit (10x Genomics, 1000265) from 10x Genomics was used to capture the transcriptome of individual cell according to the manufacturer’s protocol. ^[2]^ For each of the samples, 15,000 cells were loaded into a single well of a Chip G kit for GEM generation. Captured cells were lysed, and the released RNA was barcoded through reverse transcription from individual gel bead in the emulsion. The cDNA was then amplified for library construction, and the qualities of cDNA and cDNA libraries were assessed with an Agilent 2100 system. Finally, the libraries were sequenced on an Illumina HiSeq 10x platform (Novogene, Beijing).

Raw sequencing data files were processed using Cell Ranger software version 5.0.0 (10X Genomics) prior to subsequent analysis. This pipeline aligned the sequencing reads from fastq files to the reference genome *Mus musculus* (mm10) and quantified the expression of transcripts within each cell. Then, the analyses of these processed scRNA-seq data were carried out in *R* (version 4.1) with the package *Seurat* (version 4.0.3) from Satija Lab (https://satijalab.org/seurat/). ^[9]^ Preprocessing of scRNA-seq data was conducted as previously described. ^[2]^ Seurat objects were merged and subjected to the same quality filtering. We filtered out cells meeting any of the following criteria: gene number fewer than 200 or greater than 4000, cells with fewer than 1,000 or greater than 25,000 UMI counts and mitochondrial counts > 10%, as well as genes that were detected in less than three cells.

The *Seurat* R package was used to perform filtering, normalization, dimensionality reduction, clustering, and differential expression analysis. For each sample, counts were transformed and normalized using *SCTransform* with default thresholds, and integration of the samples was performed on the filtered and normalized objects using *Seurat*, which identifies cells that have matching biological states across datasets based on canonical correlation analysis (CCA) and utilizes mutual nearest neighbors (MNN) to correct for batch effects. Principal component analysis (PCA) was performed on the integrated object using *RunPCA*. The number of significant PCs (ndims = 30) was determined based on the *ElbowPlot* and *DimHeatmap* generated in *Seurat*. Finally, cells were clustered using the *FindNeighbors* and *FindClusters* functions. Uniform manifold approximation and projection (UMAP) was performed to project cells to 2 dimensions using the *RunUMAP* function. Cells showing coexpression of multiple cell type-specific genes were identified as doublets and removed from any downstream analysis. Module scores were evaluated using the *AddModuleScore* function in the *Seurat* package. All genes used to calculate gene scores are listed in Excel Data S1.

**Cell-cell communication analysis**

The R package *CellChat* (version 1.1.3) with default parameters was applied to study the cell-cell interactions among cell populations, as previously described. ^[10]^ In brief, initial preprocessing to identify overexpressed ligands and receptors was performed using the following functions: *identifyOverExpressedGenes*, *identifyOverExpressedInteractions,* and *projectData*. For the analysis of ligand-receptor interactions, pathway-level cell communication was calculated with *computeCommunProbPathway,* and aggregated networks were identified with *aggregateNet*. Network centrality scores were assigned with the function *netAnalysis computeCentrality.* A heatmap of differential signaling, based on significantly identified pathways from the analyses described above, was generated using the function *netAnalysis signalingRole heatmap* and restricting the visualization to the selected pathways. Finally, we classified signaling pathways and depicted conserved and context-specific pathways between anti-IgG antibody- or anti-CTLA-4 m2a antibody-treated EAM mouse hearts.

**Pseudotime analysis**

Pseudotime analyses were performed using Monocle, as described previously. ^[11]^ Briefly, the ordering was based on 1,000 genes that differed in expression between clusters selected via an unsupervised procedure: “*dpFeature*”. Then, the data space was reduced to two dimensions with the method “*DDRTree*”. Once the pseudotime was assigned for each cell, we identified genes that were significantly regulated as differentiation progressed using the “*differentialGeneTest*” function. Single cells were then ordered in pseudotime and placed along the trajectory using the “*orderCells*” function. The statistically significant threshold was set to a *P* value < 1E-04.

**Differentially expressed gene (DEG) identification and gene enrichment analysis for scRNA-seq**

DEGs were identified by the Wilcoxon Rank Sum test using the FindMarkers function in *Seurat*. DEGs were filtered using a minimum log2 FC > 0.25 and an adjusted *P* value < 0.05. Based on these DEGs, enriched GO terms were acquired using the *enrichGO* function of the clusterProfiler package following the default parameters.

**Spatial transcriptomics (ST) sequencing library preparation and sequencing**

OCT-embedded freshly frozen hearts, prepared from control, anti-IgG antibody- and anti-CTLA-4 m2a antibody-treated EAM mice, were used for the Visium Spatial Gene Expression platform by 10x Genomics (OE Biotechnology, China). The 10 μm thick horizontal sections of mouse hearts were placed on prechilled Visium Spatial Tissue Optimization slides, followed by optimization of tissue permeabilization according to the manufacturer’s protocol. We established an optimal tissue permeabilization time of 18 min for three heart sections. After H&E staining, permeabilization, reverse transcription and second-strand cDNA synthesis, Visium Spatial Single Cell 3’ Gene Expression libraries were constructed. The sequencing was performed on NovaSeq 6000 platform (S2 Cartridge, Illumina, San Diego, CA, USA).

**ST-seq data preprocessing**

Visium-prepared BCL files were demultiplexed by the SpaceRanger software (v1.2.1, 10x Genomics), followed by read alignment, tissue detection, fiducial detection, and feature-barcode matrix generation. *Mus musculus* UCSC mm10 was used for alignment. Raw counts were used as input for the *Seurat* R package, and log-normalization was implemented separately for each dataset and integrated by using the *FindIntegrationAnchors* and *IntegratedData* functions. Then, we performed a linear regression on all genes using *ScaleData*. We used *RunPCA* for dimensional reduction and FindClusters for graph-based clustering. We performed UMAP using *RunUMAP*. DEGs were detected by using *FindMarkers* in *Seurat* (log2 FC threshold > 0.25 and adjusted *P* value < 0.05).

**Deconvolution analysis**

The cell-type composition of each spot was inferred using SPOTlight (v1.5.2), as described previously. ^[12]^ SPOTlight applied a seeded nonnegative matrix factorization (NMF) regression to integrate scRNA-Seq and transcriptome data. A gene set including cell type-specific genes was used to train the SPOTlight model.

**GO term enrichment analysis DEGs in ST data**

GO term enrichment analysis was performed on differentially expressed genes using the gseapy (version 0.10.4) wrapper package, as described previously. ^[13]^ Differentially expressed genes (two-sided Wilcoxon test, log2 FC threshold = 0.5, *P*< 10^-2^ for spatial transcriptomics spots) were selected and used for GO term enrichment analysis using GO Biological Processes in the enrichr command. ^[14]^ The enriched GO terms of interest were selected and visualized on a bar plot and used to calculate module scores using the score genes command in Scanpy.

**Data and statistical analyses**

Data are presented as the mean ± SEM; *n* represents the number of biological replicates. Differences between two groups were analyzed by unpaired two-tailed Student’s *t* tests. For comparisons involving more than two groups, a one-way analysis of variance (ANOVA) followed by a post hoc Tukey test was used as appropriate. Statistical analysis was performed using the R software (version 4.1) and GraphPad Prism 7.0 software (GraphPad Software, San Diego, CA, USA). *n* is the sample size. *P* < 0.05 was considered statistically significant.


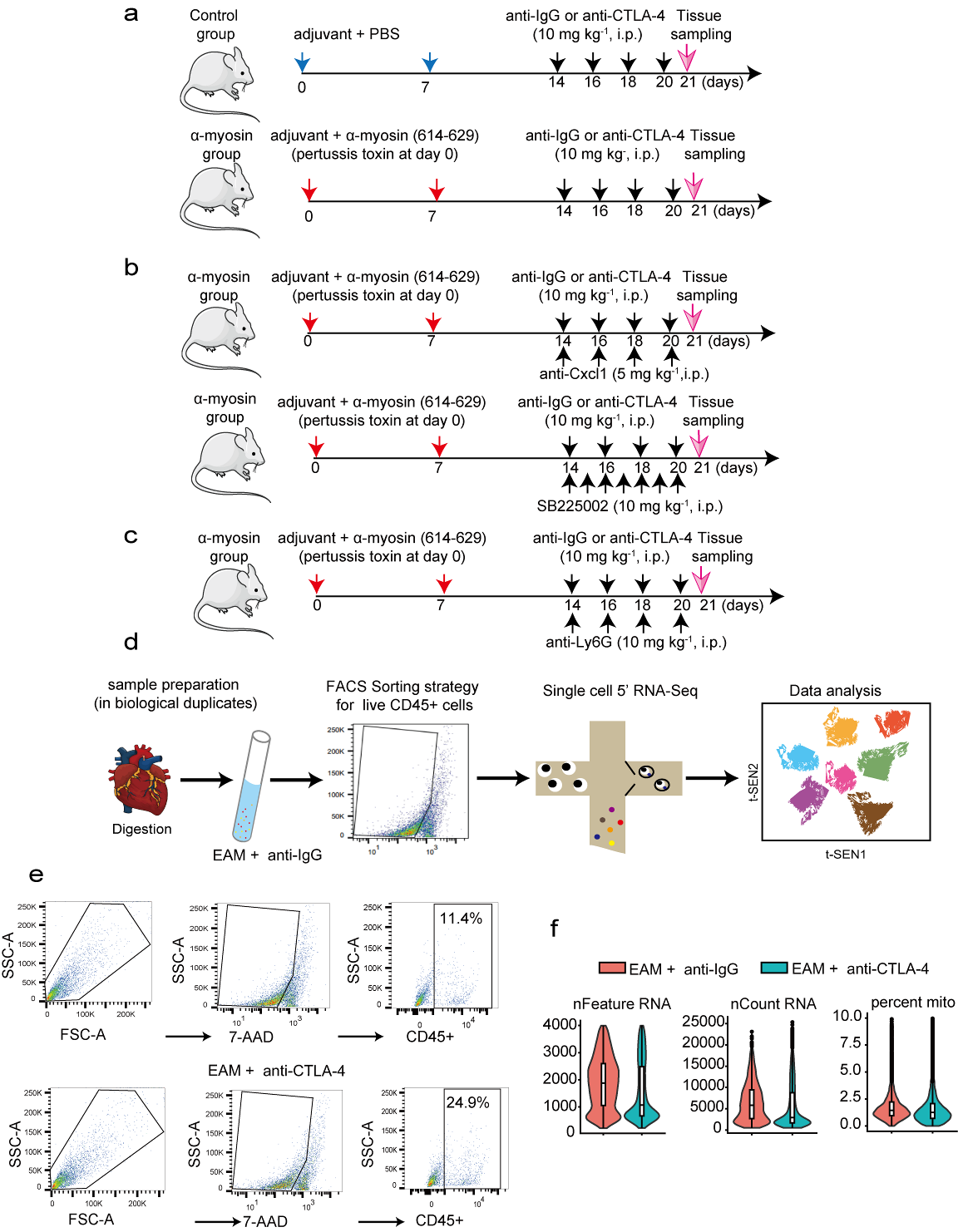


**Figure S1. Graphical illustration of the experimental setup and flow cytometry cell sorting strategy for single-cell RNA sequencing (scRNA-Seq). a-c** Schematic representation of the experimental design used in this study (refer to the Animals section of Material and Methods). **d** Single cells were respectively isolated from the hearts of anti-IgG antibody- and anti-CTLA-4 m2a antibody-treated EAM mice on the 21^st^ day post initial immunization. Hearts were digested into single-cell suspensions, and live CD45^+^7AAD^-^-cells were sorted by fluorescence-activated cell sorter (FACS) and subjected to scRNA-seq using the 10× Genomics platform. **e** Flow cytometry cell sorting strategy for scRNA-seq. CD45^+^7AAD^-^-cells were sorted from anti-IgG antibody- and anti-CTLA-4 m2a antibody-treated EAM mouse hearts. **f** After removal of doublets and low-quality cells, violin plots and box plots showing the number of features per single cell (nFeature RNA), number of unique molecular identifiers (UMIs) per single cell (nCount RNA), and percentage of mitochondrial reads per single cell (percent mito). Box plots represent the median and 25th and 75th percentiles of the cell proportion data, and whiskers represent the highest and lowest values within 1.5 times the interquartile range of the box plot.


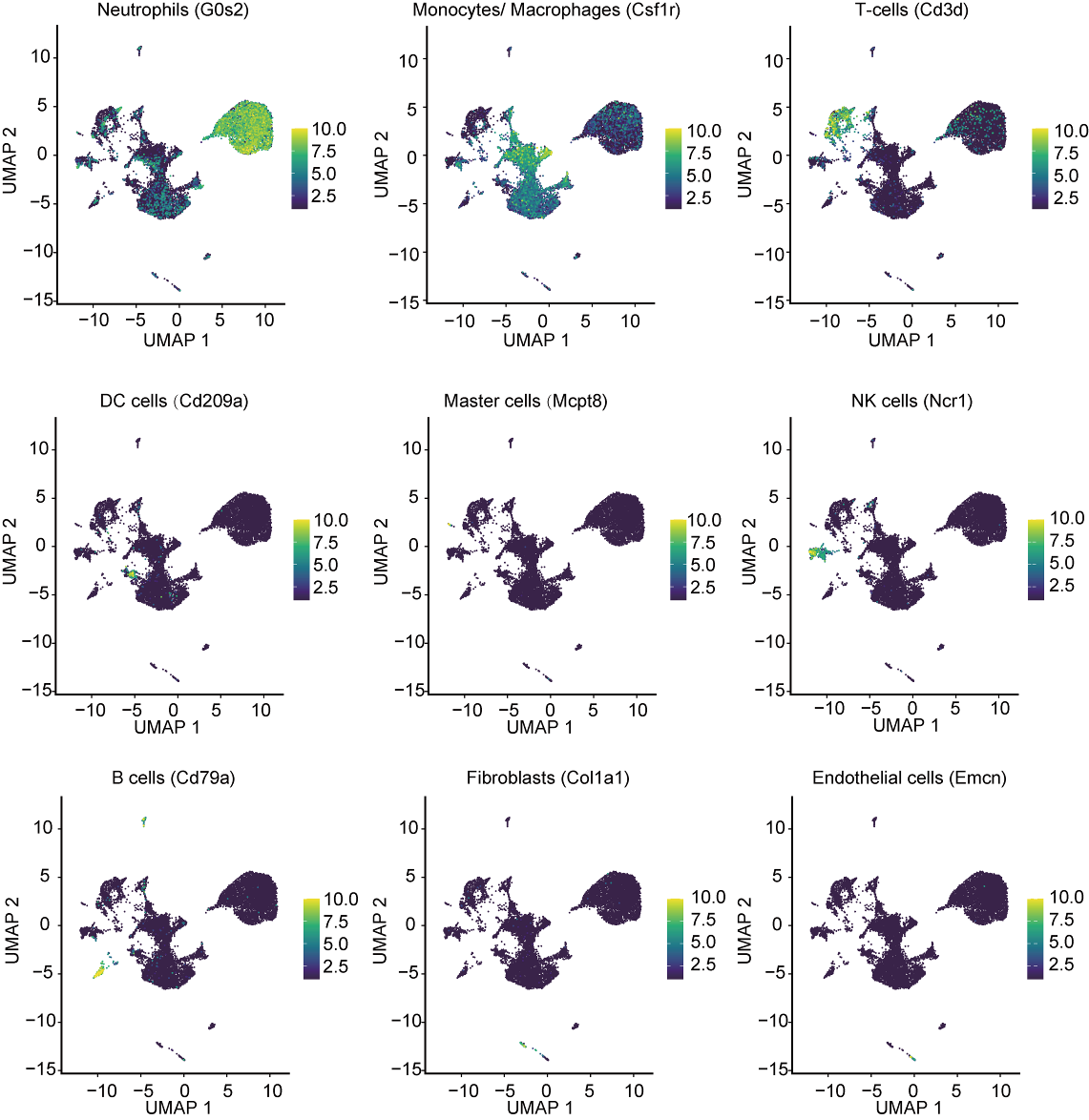


**Figure S2. Gene expression signatures of cardiac cell types.** UMAP plots showing the expression of the selected marker genes for each cell type. Each point presenting a single cell, colored according to normalized expression levels. The average expression scale shown on the right side of UMAP plot, coding from the navy to the yellow, indicates the relative gene expression levels from low to high. The top legend and marker gene indicate the specific cell type.
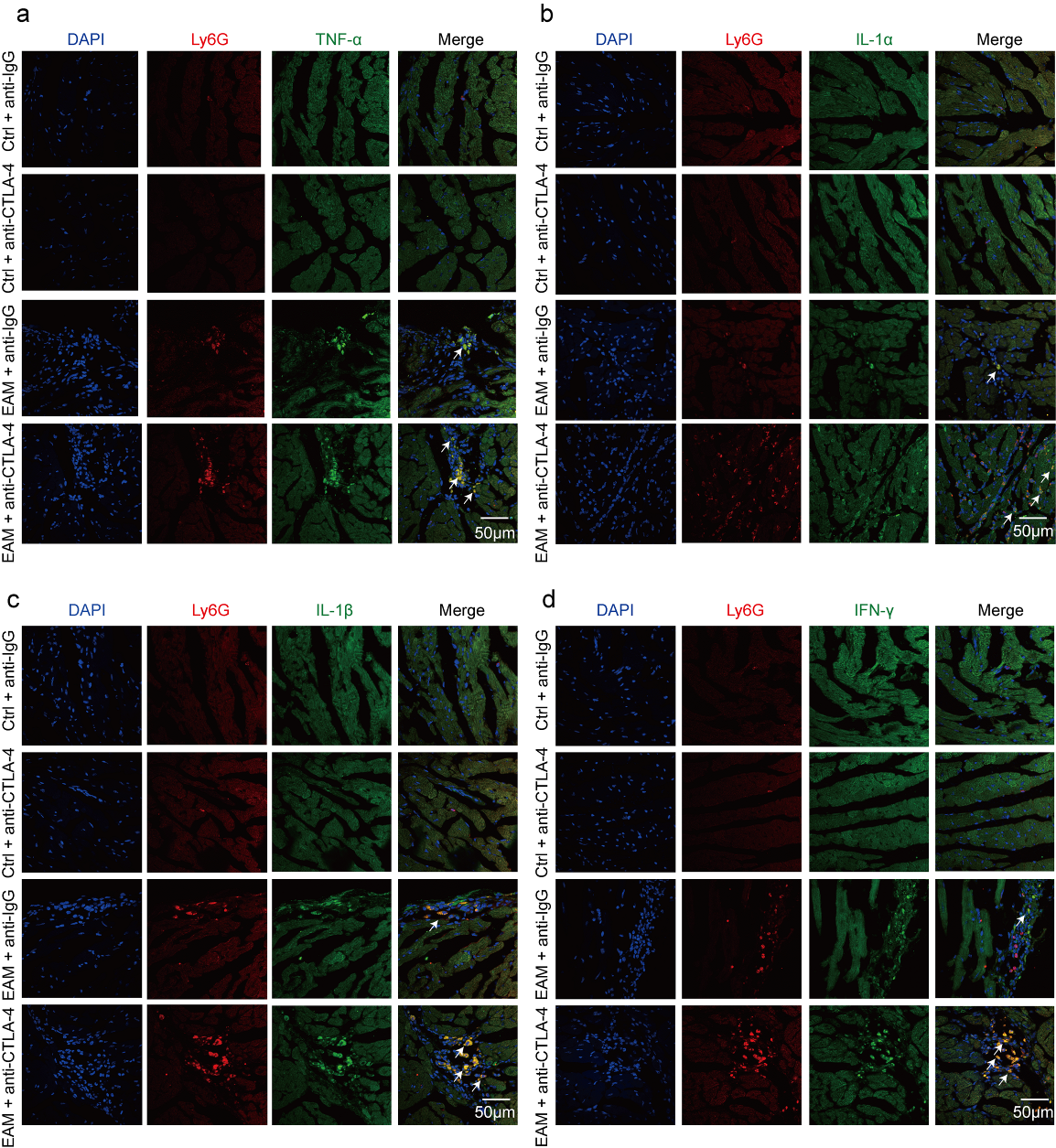


**Figure S3. a-d** Representative co-immunofluorescence staining of Ly6g (red) neutrophil with Tnf-α (**a**), Il-1α (**b**), Il-1β (**c**) and IFN-γ (**d**) (green) in the MZs of mice from each indicated experimental group. The white arrows represent the colocalization of Tnf-α, Il-1α, Il-1β and IFN-γ with the neutrophil marker Ly6G. Scale bars equal 50 μm.


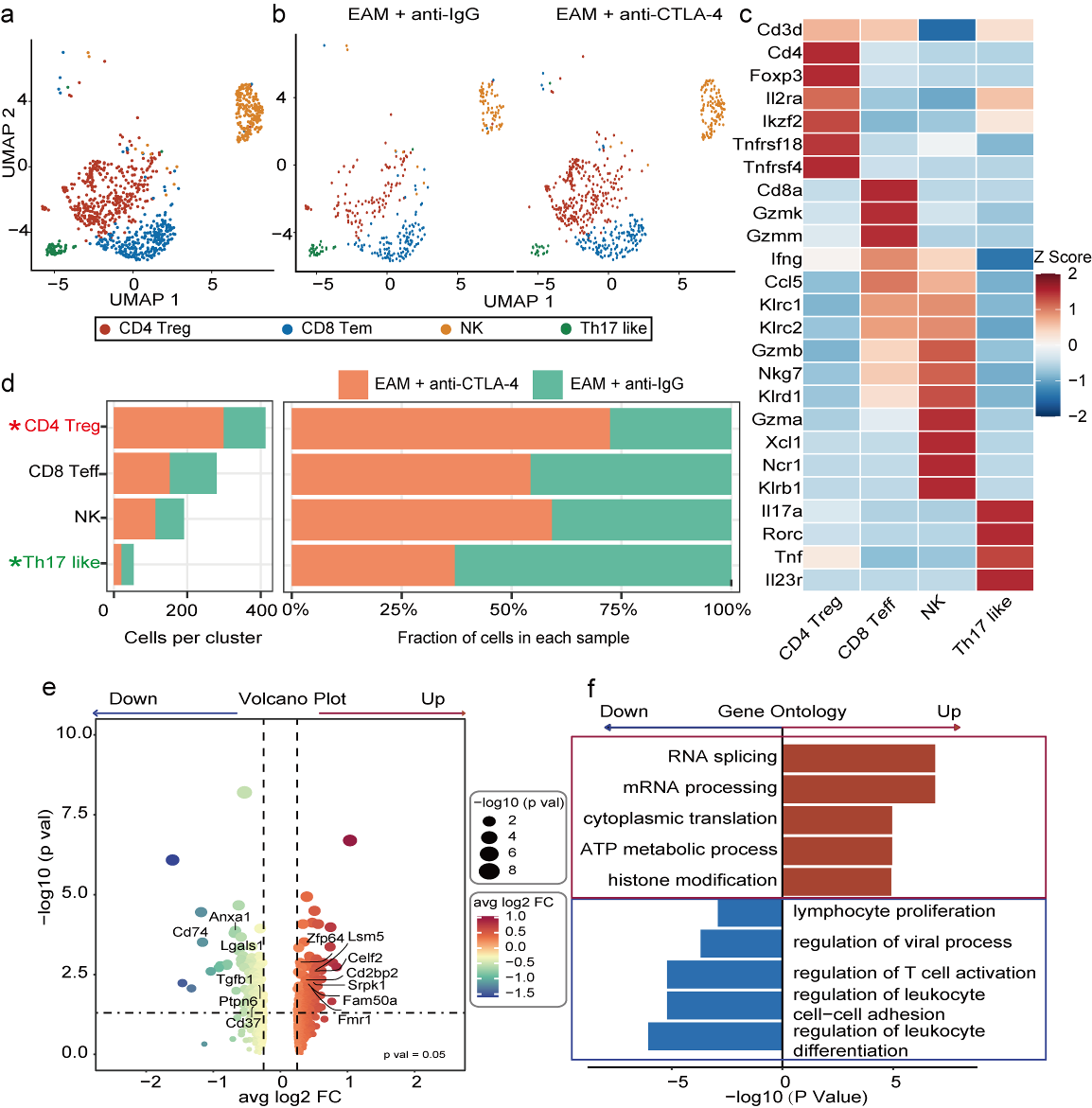


**Figure S4. Anti-CTLA-4 m2a antibody-mediated increase in the infiltration of CD4^+^ Treg cell subpopulation in the hearts of EAM mice. a** UMAP plot showing four T cell subpopulations (CD4^+^ Treg cells, CD8^+^ effector T cells, NK cells, and Th17-like cells) colored by manually annotated clusters. **b** UMAP plots demonstrating the differences of infiltrated T cell subpopulations in the hearts between anti-IgG antibody- and anti-CTLA-4 m2a antibody-treated EAM mice. **c** Heatmap showing the relative expression (as reflected by z score) levels of marker genes in each subpopulation of T cells. **d** The absolute numbers of T cell subpopulations in the heart (left panel) in each indicated experimental group and the relative proportion of T cell subpopulations scaled by the total number of cells per experimental condition (right panel); the colored bars in green and in orange respectively demonstrating the alterations in each indicated T cell subpopulation between anti-IgG antibody- and anti-CTLA-4 m2a antibody-treated EAM mice. The *P* value was calculated by the chi-squared test (the red asterisk indicates a significant increase; the green asterisk represents a significant decrease). **e** Volcano plot showing DEGs in the CD8^+^ effector T cells between anti-IgG antibody- and anti-CTLA-4 m2a antibody-treated EAM mouse hearts; the black dashed lines show the thresholds for significantly enriched genes. The depicted blue or red solid line with arrow head shown on the top respectively indicating that the expression of genes was decreased or increased by anti-CTLA-4 m2a antibody in the CD8^+^ effector T cells compared with those in anti-IgG antibody treated-EAM mice. *p* value < 0.05 and average log2FC > 0.25 were considered significantly regulated by the anti-CTLA-4 m2a antibody. As a result, 236 genes were found to be upregulated, whereas 134 genes were downregulated in the CD8^+^ effector T cells of anti-CTLA4 m2a antibody-treated EAM mouse hearts. **f** GO analysis showing that the interest GO terms for genes enriched in the CD8^+^ effector T cells were differed between anti-IgG antibody- and anti-CTLA-4 m2a antibody-treated EAM mice. Lymphocyte proliferation, regulation of viral process, regulation of T cell activation, regulation of leukocyte cell-cell adhesion, regulation of leukocyte differentiation were significantly decreased by anti-CTLA-4 m2a antibody in the CD8^+^ effector T cells of EAM mice (the blue solid rectangular bars); in contrast, RNA splicing, mRNA processing, cytoplasmic translation, ATP metabolic process, histone modification were significantly increased by anti-CTLA-4 m2a antibody in the CD8^+^ effector T cells of EAM mice (the red solid rectangular bars).


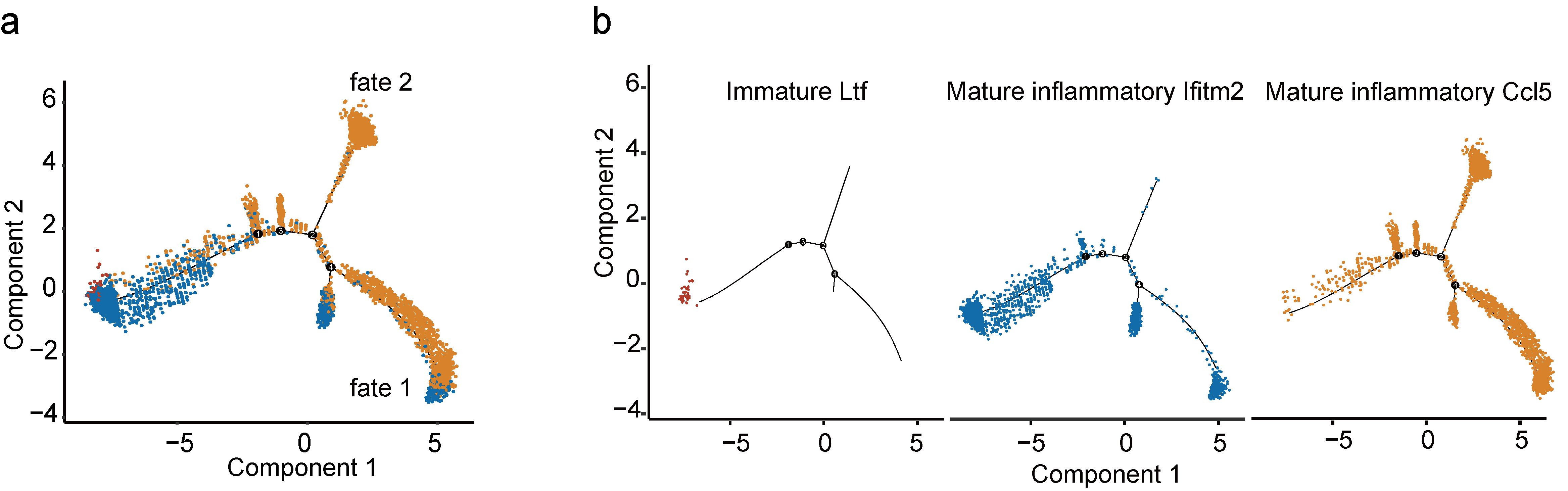


**Figure S5. Trajectory analysis of neutrophil subpopulations in the cardiac tissues.** **a** Pseudotime analysis revealed the cell trajectories within three neutrophil subpopulations. **b** Monocle pseudotime trajectories showing three distinctly distributed neutrophil subpopulations (immature Ltf-neutrophil, mature inflammatory Ifitm2-neutrophil, mature inflammatory Ccl5-neutrophil) from both anti-IgG antibody- and anti-CTLA-4 m2a antibody-treated EAM mice. Each dot represents a single cell and the dot color indicates the each indicated neutrophil subpopulation, respectively.


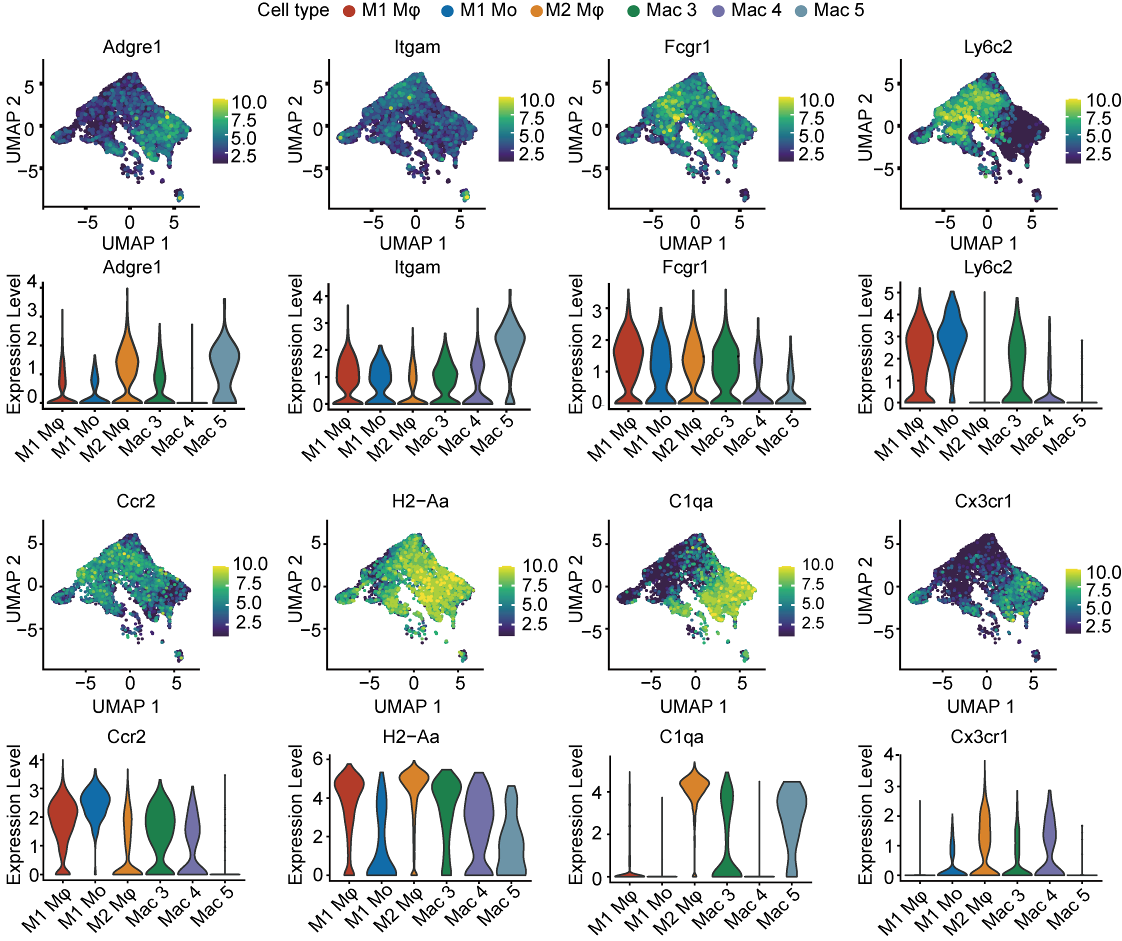


**Figure S6. Monocyte/macrophage marker gene expression in the cardiac tissues.** UMAP plots showing gene expression gradients identified within cell populations. Each point depicts a single cell, colored according to normalized expression levels (the average expression scale is shown on the right side of each UMAP plot). Feature plots and violin plots demonstrating the expression levels of marker genes Adgre1 (F4/80), Itgam (CD11b), Fcgr1 (CD64), Ly6c2, Ccr2, H2-Aa (MHC-II), C1qa, and Cx3cr1 in the annotated macrophage subpopulations. According to these marker genes, six macrophage clusters were identified (each cluster labeled with the different color) in the hearts as follows: 1) classical blood-derived M1 monocytes (M1 Mo): Adgre1(F4/80)^+^Itgam(CD11b)^+^Fcgr1(Cd64)^+^Ly6c2^high^ Ccr2^high^H2-Aa(MHC-II)^low^; 2) Classical Mo-derived M1 (M1 Mφ): Ccr2^high^Adgre1(F4/80)^+^Ly6c2^+^H2-Aa(MHC-II)^+^; 3) Nonclassical M2 macrophages (M2 Mφ): Ccr2^high^Adgre1(F4/80)^+^H2-Aa (MHC-II)^high^Ly6c2^-^; 4) Mac 3 showed upregulation of granulocyte markers, including Ccr2, Ly6c2 and H2-Aa; 5) Mac 4 subpopulations expressed markers of neutrophils (Itgam and Fcgr1) and H2-Aa; 6) Mac 5 subpopulations with lower expression (Ccr2, Cx3cr1 and Ly6c2), and higher expression of C1qa.


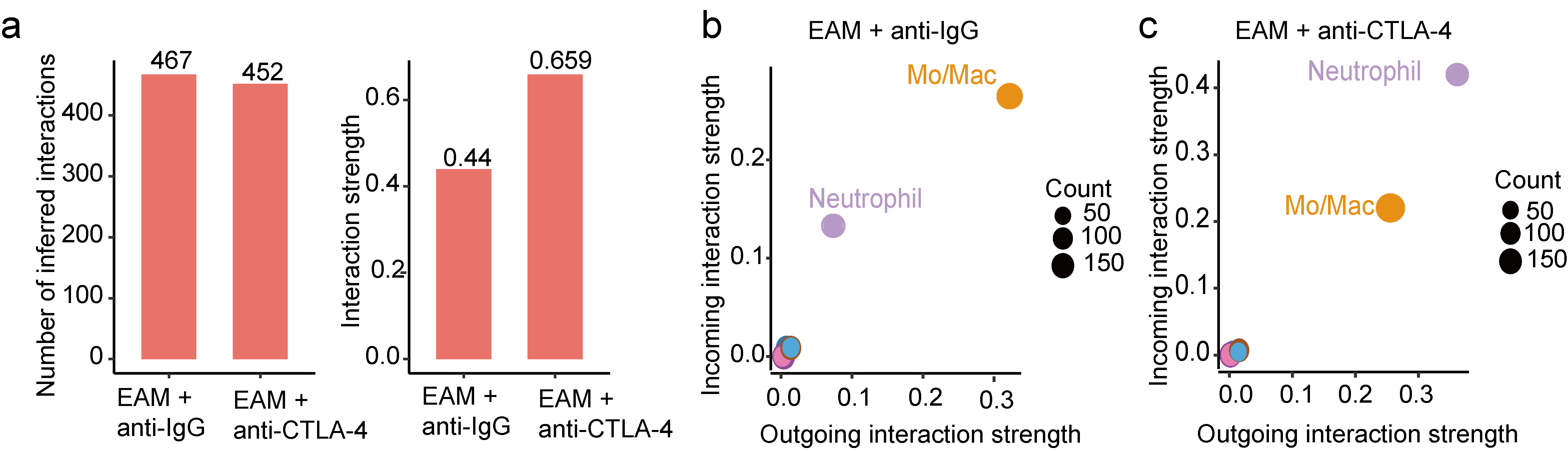


**Figure S7. Cell-cell communication analyses in anti-IgG antibody- and anti-CTLA-4 m2a antibody-treated EAM mouse hearts.** **a** Bar graphs showing the total number of interactions and interaction strength of the inferred cell-cell communication networks in anti-IgG antibody- and anti-CTLA-4 m2a antibody-treated EAM mouse hearts. **b**, **c** Scatter plots showing the outgoing and incoming interaction strength of each cell type in anti-IgG antibody- (**b**) and anti-CTLA-4 m2a antibody-treated EAM mouse hearts (**c**).


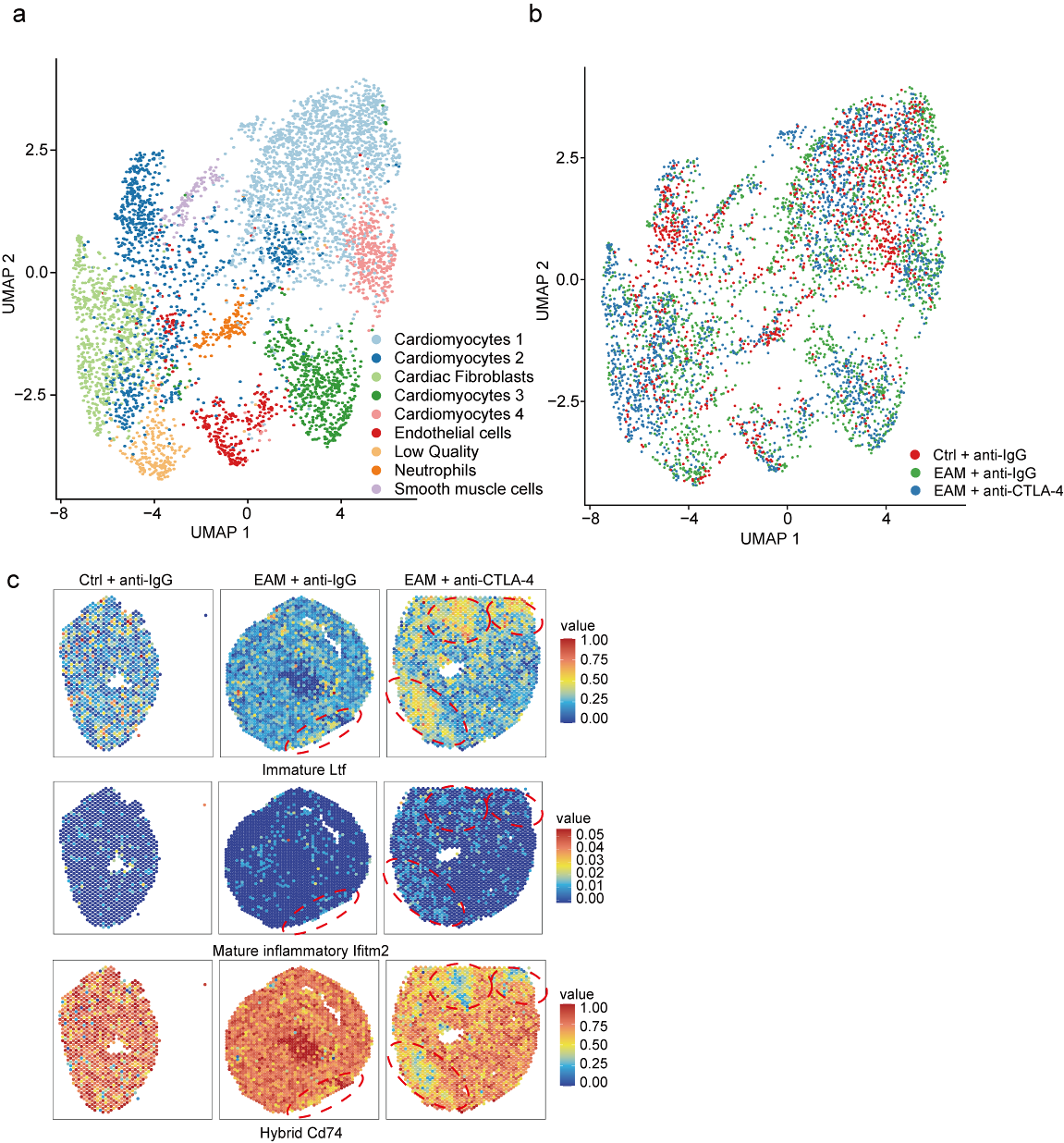


**Figure S8. Identifies cell types by spatial transcriptomics analysis in cardiac tissue. a** UMAP showing the cell-type spots on the cardiac sections prepared from each indicated experimental group. All spots (dots) were classified into nine clusters (Cardiomyocytes 1, Cardiomyocytes 2, Cardiac fibroblasts, Cardiomyocytes 3, Cardiomyocytes 4, Endothelial cells, Low-quality cells, Neutrophils, Smooth muscle cells). **b** UMAP plots showing the distribution of the cells (spots) in cardiac tissue of three indicated experimental groups (control mice, anti-IgG antibody- and anti-CTLA-4 m2a antibody-treated EAM mice). **c** SPOTlight mapping on Visium ST from the cardiac sections demonstrating that localization of immature Ltf-neutrophil subpopulation (upper panel), mature inflammatory Ifitm2-neutrophil subpopulation (middle panel) and hybrid Cd-74-neutrophil subpopulation (lower panel) in the myocarditic zones (MZs). The colored bars, shown on the right of each panel, indicate the relative abundance of the immature Ltf-neutrophil subpopulation, mature inflammatory Ifitm2-neutrophil subpopulation, and hybrid Cd-74-neutrophil subpopulation from low (the blue) to high (the red) (circled by the dashed red lines).


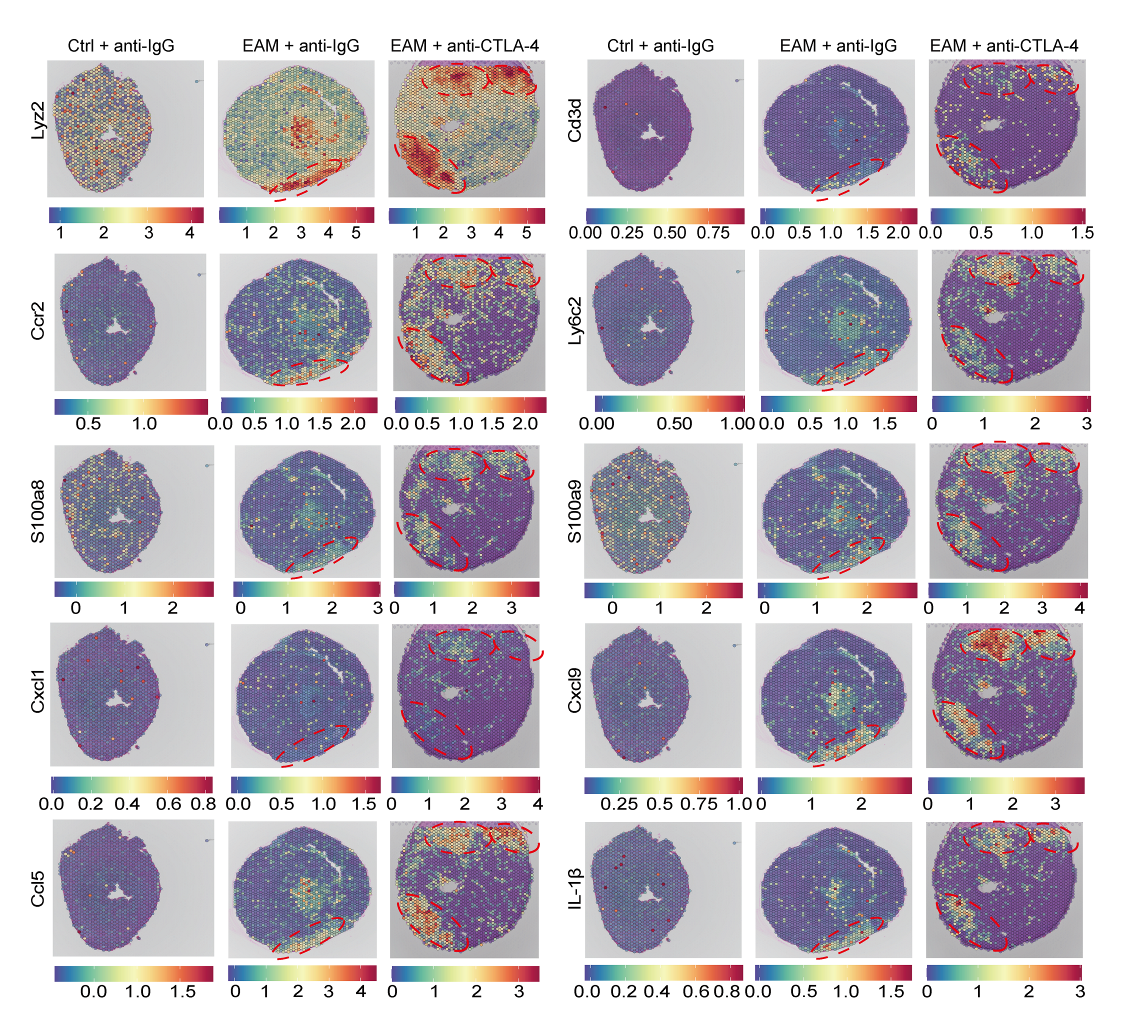


**Figure S9. The transcriptomic profiles and cell-type-specific signatures in MZs.** Spatial feature plots showing the expression levels of the representative genes Lyz2, Cd3d, Ccr2, Ly6c2, S1000a8, S100a9, Cxcl1, Cxcl9, CCl5, and IL-1β in the MZs of each indicated experimental group. The colored bars shown on the bottom of each panel, coding from the blue to the red, indicate the relative gene expression levels from low to high.


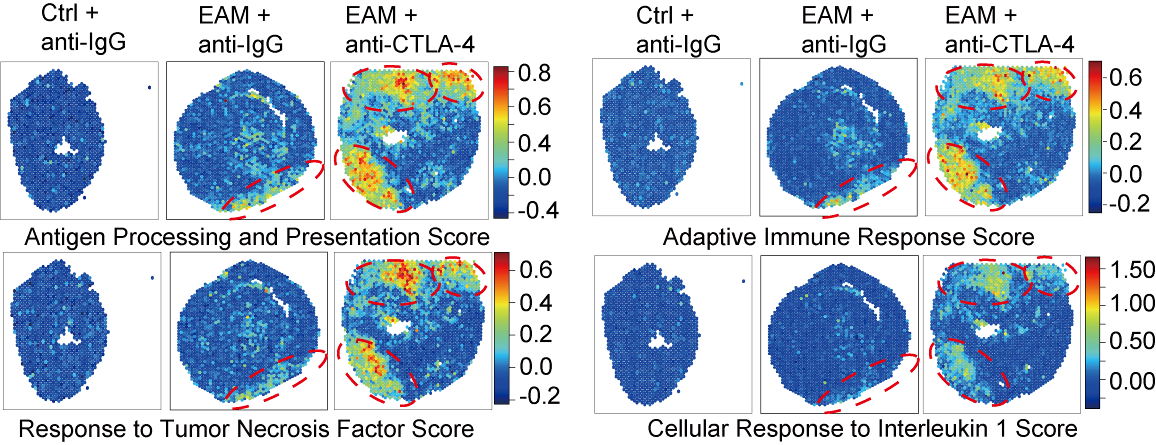


**Figure S10. The immune/proinflammatory cytokine scores were calculated for the interest GO terms enriched in MZs.** ST maps showing that calculated GO terms scores for four interested immune/proinflammatory cytokines signaling (antigen processing and presentation, adaptive immune response, response to tumor necrosis factor, and cellular response to interleukin 1) were dramatically enriched by anti-CTLA-4 m2a antibody in the MZs of EAM mice (circled by the dashed red lines). The colored bars shown on the right of each panel, coding from the blue to the red, indicated the signaling activity scores from low to high.


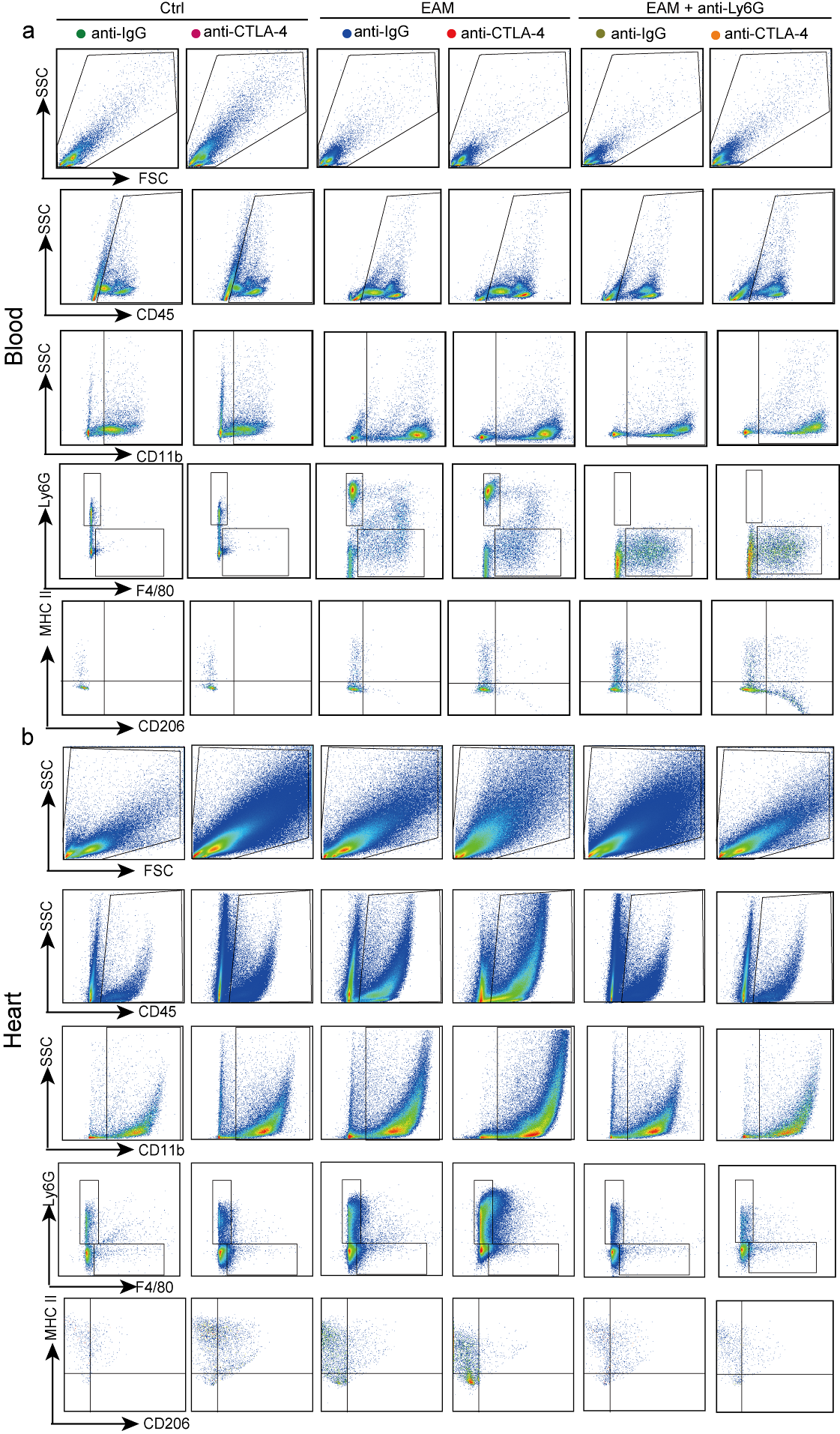


**Figure S11. Representative flow cytometry and gating strategy.** The representative flow cytometry dot plots, obtained from the peripheral blood (**a**) and cardiac tissues (**b**) of each indicated experimental group, showing the gating strategies for identifying CD45^+^CD11b^+^Ly6G^+^ (neutrophil), CD45^+^CD11b^+^F4/80^+^ (Mφ), CD45^+^CD11b^+^F4/80^+^MHCII^+^ (M1 Mφ) and CD45^+^CD11b^+^F4/80^+^CD206^+^ (M2 Mφ).

**Supplementary Table 1.** Oligonucleotide primers for qRT-PCR.

| Gene | Forward (5’ > 3’) | Reverse (5’ > 3’) |
| --- | --- | --- |
| Cxcl1 | GCCTATCGCCAATGAGCTG | GCATCTTTTGGACAATTTTCTG |
| IL-1α | TCGGGAGGAGACGACTCTAA | TGGATAAGCAGCTGATGTGAA |
| IL-1β | GGGCCTCAAAGGAAAGAATC | CTCTGCTTGTGAGGTGCTGA |
| INF-γ | GTACAACCTCCTTGCAGCTCCT | TTGTCGACGACGAGCGC |
| TNF-α | CGTCAGCCGATTTGCTATCT | CGGACTCCGCAAAGTCTAAG |
| GAPDH | ATTGTTGCCATCAACGACCC | CCACGACATACTCAGCACC |

**References**

[1] N. Percie du Sert, V. Hurst, A. Ahluwalia, S. Alam, M. T. Avey, M. Baker, W. J. Browne, A. Clark, I. C. Cuthill, U. Dirnagl, M. Emerson, P. Garner, S. T. Holgate, D. W. Howells, N. A. Karp, S. E. Lazic, K. Lidster, C. J. MacCallum, M. Macleod, E. J. Pearl, O. H. Petersen, F. Rawle, P. Reynolds, K. Rooney, E. S. Sena, S. D. Silberberg, T. Steckler, H. Wurbel, *PLoS Biol* **2020**, *18* (7), e3000410, <https://doi.org/10.1371/journal.pbio.3000410>.

[2] X. Hua, G. Hu, Q. Hu, Y. Chang, Y. Hu, L. Gao, X. Chen, P. C. Yang, Y. Zhang, M. Li, J. Song, *Circulation* **2020**, *142* (4), 384, <https://doi.org/10.1161/CIRCULATIONAHA.119.043545>.

[3] a) S. J. O'Day, M. Maio, V. Chiarion-Sileni, T. F. Gajewski, H. Pehamberger, I. N. Bondarenko, P. Queirolo, L. Lundgren, S. Mikhailov, L. Roman, C. Verschraegen, R. Humphrey, R. Ibrahim, V. de Pril, A. Hoos, J. D. Wolchok, *Ann Oncol* **2010**, *21* (8), 1712, <https://doi.org/10.1093/annonc/mdq013>; b) M. J. Selby, J. J. Engelhardt, M. Quigley, K. A. Henning, T. Chen, M. Srinivasan, A. J. Korman, *Cancer Immunol Res* **2013**, *1* (1), 32, <https://doi.org/10.1158/2326-6066.CIR-13-0013>; c) F. Martins, L. Sofiya, G. P. Sykiotis, F. Lamine, M. Maillard, M. Fraga, K. Shabafrouz, C. Ribi, A. Cairoli, Y. Guex-Crosier, T. Kuntzer, O. Michielin, S. Peters, G. Coukos, F. Spertini, J. A. Thompson, M. Obeid, *Nat Rev Clin Oncol* **2019**, *16* (9), 563, <https://doi.org/10.1038/s41571-019-0218-0>.

[4] H. Li, M. Zhang, Q. Zhao, W. Zhao, Y. Zhuang, J. Wang, W. Hang, Z. Wen, L. Wang, C. Chen, D. W. Wang, *Cell Discov* **2023**, *9* (1), 103, <https://doi.org/10.1038/s41421-023-00593-5>.

[5] Y. S. Hwang, H. J. Cho, E. S. Park, J. Lim, H. R. Yoon, J. T. Kim, S. R. Yoon, H. Jung, Y. K. Choe, Y. H. Kim, C. H. Lee, Y. T. Kwon, B. Y. Kim, H. G. Lee, *Cells* **2022**, *11* (24), <https://doi.org/10.3390/cells11244101>.

[6] C. J. Yu, C. Liang, Y. X. Li, Q. Q. Hu, W. W. Zheng, N. Niu, X. Yang, Z. R. Wang, X. D. Yu, B. L. Zhang, B. L. Song, Z. R. Zhang, *Hypertension* **2017**, *69* (4), 615, <https://doi.org/10.1161/HYPERTENSIONAHA.116.08500>.

[7] J. Schindelin, I. Arganda-Carreras, E. Frise, V. Kaynig, M. Longair, T. Pietzsch, S. Preibisch, C. Rueden, S. Saalfeld, B. Schmid, J. Y. Tinevez, D. J. White, V. Hartenstein, K. Eliceiri, P. Tomancak, A. Cardona, *Nat Methods* **2012**, *9* (7), 676, <https://doi.org/10.1038/nmeth.2019>.

[8] M. M. Wu, C. Liang, X. D. Yu, B. L. Song, Q. Yue, Y. J. Zhai, V. Linck, Y. X. Cai, N. Niu, X. Yang, B. L. Zhang, Q. S. Wang, L. Zou, S. Zhang, T. L. Thai, J. Ma, R. L. Sutliff, Z. R. Zhang, H. P. Ma, *Br J Pharmacol* **2019**, *176* (18), 3695, <https://doi.org/10.1111/bph.14775>.

[9] Y. Hao, S. Hao, E. Andersen-Nissen, W. M. Mauck, 3rd, S. Zheng, A. Butler, M. J. Lee, A. J. Wilk, C. Darby, M. Zager, P. Hoffman, M. Stoeckius, E. Papalexi, E. P. Mimitou, J. Jain, A. Srivastava, T. Stuart, L. M. Fleming, B. Yeung, A. J. Rogers, J. M. McElrath, C. A. Blish, R. Gottardo, P. Smibert, R. Satija, *Cell* **2021**, *184* (13), 3573, <https://doi.org/10.1016/j.cell.2021.04.048>.

[10] a) N. Lasrado, N. Borcherding, R. Arumugam, T. K. Starr, J. Reddy, *iScience* **2022**, *25* (3), 103865, <https://doi.org/10.1016/j.isci.2022.103865>; b) S. Jin, C. F. Guerrero-Juarez, L. Zhang, I. Chang, R. Ramos, C. H. Kuan, P. Myung, M. V. Plikus, Q. Nie, *Nat Commun* **2021**, *12* (1), 1088, <https://doi.org/10.1038/s41467-021-21246-9>.

[11] C. Trapnell, D. Cacchiarelli, J. Grimsby, P. Pokharel, S. Li, M. Morse, N. J. Lennon, K. J. Livak, T. S. Mikkelsen, J. L. Rinn, *Nat Biotechnol* **2014**, *32* (4), 381, <https://doi.org/10.1038/nbt.2859>.

[12] M. Elosua-Bayes, P. Nieto, E. Mereu, I. Gut, H. Heyn, *Nucleic Acids Res* **2021**, *49* (9), e50, <https://doi.org/10.1093/nar/gkab043>.

[13] M. Mantri, M. M. Hinchman, D. W. McKellar, M. F. Z. Wang, S. T. Cross, J. S. L. Parker, I. De Vlaminck, *Nat Cardiovasc Res* **2022**, *1* (10), 946, <https://doi.org/10.1038/s44161-022-00138-1>.

[14] Z. Xie, A. Bailey, M. V. Kuleshov, D. J. B. Clarke, J. E. Evangelista, S. L. Jenkins, A. Lachmann, M. L. Wojciechowicz, E. Kropiwnicki, K. M. Jagodnik, M. Jeon, A. Ma'ayan, *Curr Protoc* **2021**, *1* (3), e90, <https://doi.org/10.1002/cpz1.90>.
